# Educational interventions on fever management in children: A scoping review

**DOI:** 10.1002/nop2.294

**Published:** 2019-05-01

**Authors:** Daniel Arias, Timothy F. Chen, Rebekah J. Moles

**Affiliations:** ^1^ School of Pharmacy The University of Sydney Sydney NSW Australia

**Keywords:** children, education, fever, fever management, intervention, nurse, paediatrics, pharmacist, physician, scoping review

## Abstract

**Background:**

Numerous studies have been conducted specifically to target “fever phobia” and inappropriate fever management skills. However, despite educational intervention, caregivers continue to adopt inappropriate and non‐evidence‐based practices.

**Aims:**

To collect and examine peer‐reviewed literature for active educational interventions aimed at improving fever management in children and profile them based on: who provided the training, training location, how the intervention was delivered, outcomes of training, and how it was measured.

**Design:**

Scoping Review.

**Methods:**

MEDLINE, EMBASE, CINAHL, PubMED, PsycINFO, and IPA were searched from January 1980–December 2016. Study location, type of intervention, intervention target, study aim(s), sample size, instruments, outcome measures, and results were extracted.

**Results:**

Thirty‐seven studies met the inclusion criteria. Most targeted parents with the remainder focused on healthcare professionals. The interventions and their outcome measures varied significantly from structured group training sessions to video interventions and many using a combination of methods. Most interventions reported a positive impact in outcomes such as knowledge, health service use, or fever management skills.

**Conclusion:**

More standardized educational platforms targeted at both caregivers and healthcare professionals with appropriate evaluation methods should be developed and made widely available.

## 1 INTRODUCTION

While body temperature varies, normal is considered to be between 36 and 37.5°C (NSWHealth, 2010). Fever is defined as core body temperatures greater than 38°C (Kluger, 1979; NSWHealth, 2010). Fever is a biological response to invading infections and a beneficial host defence mechanism (El‐Radhi, 2012; Soszynski, 2003). Fever is common in children, and all caregivers will need to manage a feverish child at some stage. However, fever is often inappropriately managed causing safety concerns (Crocetti, Moghbeli, & Serwint, 2001). A recent study showed that fever accounted for 20% of paediatric emergency room (ER) visits (Baker, Monroe, King, Sorrentino, & Glaeser, 2009). Further, 82% of ER presentations were classified as non‐urgent and more appropriately managed at home (Berry, Brousseau, Brotanek, Tomany‐Korman, & Flores, 2008).

The overuse of healthcare services stems from both caregivers and healthcare providers viewing fever as a sign of severe underlying illness and treating it as a disease, rather than a symptom (El‐Radhi, 2008). This inherent exaggerated fear has been coined “fever phobia” (Abdullah, Ashong, Al Habib, Karrar, & Al Jishi, 1987; Schmitt, 1980). Caregivers fear that untreated fever leads to harmful effects such as febrile seizures, brain damage, and death (Blumenthal, 1998). These fears lead to overtreatment and overuse of public health care (Richardson & Purssell, 2015). Similarly, healthcare providers also harbour misconceptions (Demir & Sekreter, 2012; May & Bauchner, 1992) and may add to anxiety of caregivers. One study showed that up to 65% of physicians indicated that fever was harmful and 90% believed that febrile convulsions could cause brain damage (Demir & Sekreter, 2012).

Most of this fear is from lack of knowledge and education (Blumenthal, 1998) leading to non‐evidence‐based management (Zyoud et al., 2013). While international guidelines have been implemented (Baker et al., 2009; Chiappini et al., 2013; National Collaborating Centre for Women's and Children's Health (UK), 2013; NSWHealth, 2010; SAHealth, 2013), many physicians still debate fever harms and treatments (El‐Radhi, 2012). A recent study by Raffaeli et al. (2016), evaluating views and practices of healthcare providers, found that over 30% could not correctly define a fever and many did not know recommended doses of antipyretics (Raffaeli et al., 2016). Shakeel, Iffat, and Qamar (2014) found most physicians recommending inappropriate physical methods to lower children's fever including baths (90.14%), cold applications (82.39%), and rubbing the body with alcohol (28.87%) (Shakeel et al., 2014).

This creates a difficult obstacle, as fever management skills of caregivers may be informed by healthcare providers, friends, family beliefs, the Internet, or written literature (So & Moles, 2014; Walsh, Edwards, & Fraser, 2008). Information originating from multiple sources can be highly conflicting and can cause increased uncertainty (Walsh et al., 2008). Some common errors include the following: not taking temperatures; relying on temperature measurements independent of symptoms; using physical means such as sponging or bathing; and inappropriate use of medicines including incorrect doses, dosing intervals, or combinations of treatments (So & Moles, 2014). Numerous studies have been conducted specifically to target “fever phobia” and inappropriate fever management skills. Interventions directed at caregivers were found to target different concerns such as reducing fever anxiety (O'Neill‐Murphy, Liebman, & Barnsteiner, 2001), increasing the amount of information given to parents (Considine & Brennan, 2007), or focusing on measuring and improving knowledge (Emmerton et al., 2014). Despite educational intervention, caregivers continue to adopt inappropriate and non‐evidence‐based practices (Chiappini et al., 2013; Monsma, Richerson, & Sloand, 2015; O'Neill‐Murphy et al., 2001; So & Moles, 2014; Zyoud et al., 2013).

The aim of fever management is to protect and comfort the child until a diagnosis of the underlying condition is made (National Collaborating Centre for Women's and Children's Health (UK), 2013). Caregivers can often manage their child's temperature at home with regular fluids and rest. Worrying symptoms include the following: skin colour or texture changes; rash; drowsiness and breathing difficulty; and detection of fever in very young children (<3 months). These symptoms certainly warrant further medical attention (National Collaborating Centre for Women's and Children's Health (UK), 2013). Antipyretics should not be used with the purpose of lowering temperature but merely to comfort a child with pain associated with fever (Hewson, 2000). While guidelines are available outlining how to correctly manage children's fever, “fever phobia” is a persistent issue. Adherence of healthcare providers to new guidelines could make a huge impact in dissemination of up‐to‐date evidence‐based information (Chiappini et al., 2013; Crocetti et al., 2001); however, identifying and overcoming local barriers is essential in changing healthcare provider's behaviours to adopt and implement such guidelines (Grimshaw et al., 2004).

A literature review by Walsh and Edwards (2006) aimed to understand caregiver's attitudes, practices, and behaviours regarding treatments, medication dosing, and information seeking of caregivers. The study concluded that despite the previous success of many educational interventions, many caregiver's attitudes and practices did not change long term and interventions that targeted behavioural change and correcting caregiver influences were necessary (Walsh & Edwards, 2006). Following this, Young, Watts, and Wilson (2010) supported the notion that behavioural change is necessary to improve fever management outcomes in parents and concluded that formal education including mixed methods in either structured or repeated sessions was most effective in improving parental knowledge. In addition, a review by Monsma et al. (2015) looked at factors that should be considered when designing educational interventions aimed at caregivers and recommended that interventions that were structured, one‐on‐one and reinforced over time would provide the most effective fever management interventions.

The aim of this scoping review was to collect and examine the peer‐reviewed literature for all active educational interventions aimed at improving fever management in children and profile them based on: who provided the training, where the training took place, how the intervention was delivered, the outcomes of the training, and how they were measured. A collation of this information allows us to try to ascertain effective methods to teach fever management.

## 2 DESIGN

A scoping review was chosen, due to the heterogeneity of fever educational interventions.

### 2.1 Search methods

Articles written in English aimed at human patients from January 1980–December 2016 were identified using MEDLINE, EMBASE, CINAHL, PubMED, PsycINFO, and International Pharmaceutical Abstracts (IPA). Search strategy and keywords can be viewed in Appendix S1 and S2. Hand searches of references in included articles were also undertaken.

### 2.2 Analysis

**Figure 1:**
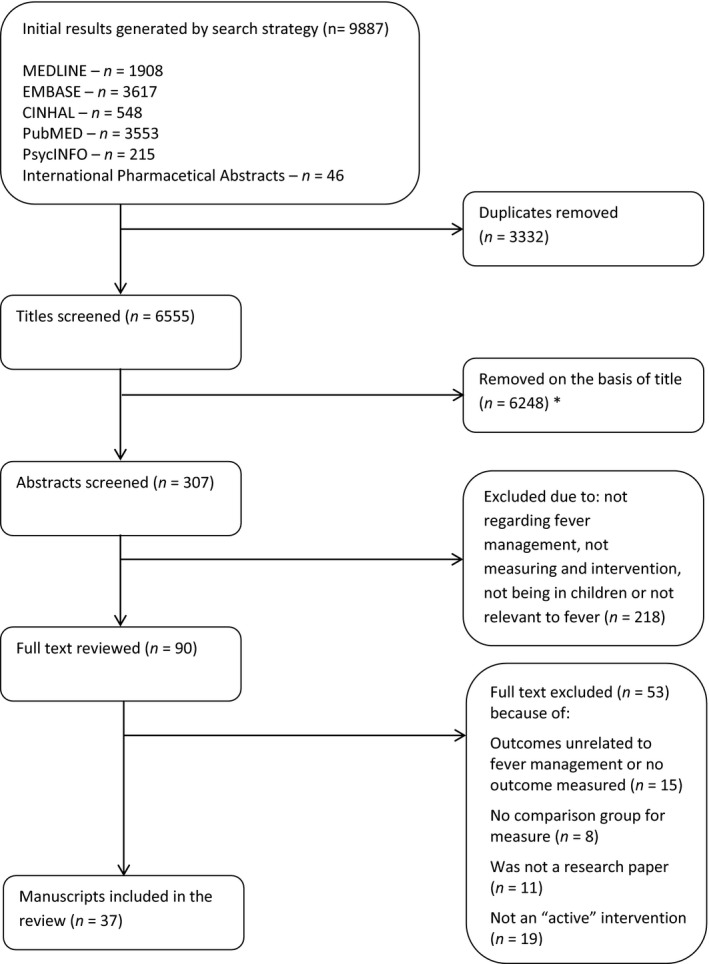
Flow chart following search strategy and study selection based on the PRISMA guidelines. ^*^Removed all articles not concerning fever or management

One author (D.A.) screened titles and abstracts, and from those deemed relevant, full articles were obtained and reported in accordance with PRISMA guidelines (Moher, Liberati, Tetzlaff, & Altman, 2009) (Figure 1). All three authors (DA, TC, and RM) met regularly to apply specific study inclusion and exclusion criteria. Studies were included if the primary focus was on an active educational intervention which incorporated improving fever management skills for children and included at least one outcome measure for evaluating the educational intervention. For the purpose of this review, an educational intervention was defined as a tool, activity, simulation, or discussion. An “active” educational intervention was defined as a model of delivering the information where the participant was taught the information by a third party. This included all audio, video, presentation, lecture/seminar/tutorial, one‐on‐one, peer‐to‐peer, demonstration, and computer‐guided interventions. Studies which involved an intervention not defined as “active” were excluded such as those requiring participants to take self‐directed initiative without external aid, including but not limited to paper‐based written materials such as guidelines, pamphlets, and posters.

Educational interventions aimed at all trained or untrained participants, caregivers/parents, and students or healthcare professionals were included. Similarly, all interventions were included regardless of training style, location, country of origin, or timeframe of the study. Data were included regardless of the level of bias or quality of the intervention.

Studies which detailed an educational intervention covering multiple topics areas not limited to children's fever were included if the intervention contained content and outcome on improving children's fever management. Studies, which covered an educational intervention concerning a disease or issues whose primary manifestation were children's fever, were included providing that they included fever management in these scenarios. This included interventions with a focus on urinary tract infections (UTIs), serious bacterial infections (SBIs), and malaria. In addition, studies which included educational interventions created as a proof of concept with no intervention and only participant satisfaction as an outcome was excluded. All manuscripts that were not primary research papers including secondary texts, literature reviews, conferences, editorials, abstracts, and posters were excluded.

### 2.3 Data abstraction

Author; year of publication; study design; location of study; type of intervention; target of intervention; study aim(s), sample size used; measurement instruments; outcome measures; and results were extracted from each manuscript.

### 2.4 Search outcome

The search strategy identified 9,887 articles. After removing duplicates and applying inclusion criteria, a total of 37 manuscripts were reviewed (Figure 1). A reference list of all reviewed manuscripts is found in Appendix S3. It should be noted that three studies by Considine et al. (2007) [S9–S11] and 2 studies by Edwards H. et al. (2007) [S15, S16] collected data from the same samples. However, the objective in each manuscript was disparate enough for these studies to be considered as separate studies for the purpose of this review.

### 2.5 Quality appraisal

Study bias was assessed using either the STROBE checklist (von Elm et al., 2007) or Cochrane checklist for randomized control trials (Higgins et al., 2011) where applicable and was rated using a low–high scale. All manuscripts were also mapped to Miller's framework for clinical competency to extrapolate whether participants demonstrated knowledge, competence, performance, or action following intervention (Miller, 1990). Further, data were evaluated for constructive alignment of the teaching method and assessment used (Biggs, 2003).

### 2.6 Ethics approval

Ethics approval was not required for this review.

## 3 RESULTS

### 3.1 Study designs

Of the 37 reviewed manuscripts (Appendix S3), there were nine randomized control trials (RCTs) [S3, S4, S14, S17, S19, S21, S23, S29, S30], twenty‐one pre–post studies [S6–S13, S15, S16, S18, S20, S22, S24, S27, S28, S31–S33, S35, S36], two descriptive studies [S25, S26], two cross‐sectional studies [S1, S37], two cohort studies [S2, S5], and one study had an interrupted time series design [S34].

### 3.2 Who was training aimed at?

Nineteen interventions were targeted at parents and caregivers [S2–S6, S8, S12, S18, S19, S21–S23, S25, S26, S28, S30, S32, S33, S36], six targeted nurses [S9–S11, S15, S16, S24], three targeted healthcare staff including clinicians, nurses, and pharmacists [S7, S14, S31], two targeted physicians only [S13, S34], three targeted healthcare workers in Africa (using a train the trainer method) [S1, S17, S37], one targeted shopkeepers [S27], one targeted caregivers and nurses [S20], and two targeted medical students [S29, S35].

With respect to caregivers, mothers were the most common participants (80% = 1,884/2,350).

### 3.3 Where was the training conducted?

Twelve interventions were conducted in hospitals [S5, S9–S11, S15, S16, S21, S23, S24, S31–S32, S35], eight were in emergency departments [S2, S3, S14, S19, S20, S28, S29, S34], six were in public health facilities [S1, S4, S7, S8, S17, S37], five at primary health practices/clinics [S13, S18, S25, S33, S36], one at a community centre [S12], one at an after‐hours clinic [S30], one at the shopkeeper's store [S27], one used a Nursing Triage Hotline database [S26], and two locations were unspecified [S6, S22].

### 3.4 Types of educational interventions

Eleven educational interventions were structured as group training sessions including lectures, discussions, tutorials, demonstrations, and organized modules [S1, S6, S8, S13, S14, S17, S18, S21, S22, S35, S37]; five used a video intervention [S2–S4, S23, S30]; six used peer‐to‐peer education session/s [S9–S11, S15, S16, S31]; five interventions were one‐on‐one sessions with either a researcher or an educated teacher in the field [S5, S25, S28, S33, S36]; three manuscripts researched a combination of group training, one‐on‐one sessions, and material dissemination [S7, S12, S27]; four used computerized training tutorials and guidelines [S19, S29, S32, S34]; one reviewed a nursing triage hotline protocol [S26]; one was a comparison between blended learning (2 face‐to‐face + 3 online sessions) and face‐to‐face learning (5 sessions) [S24]; and one used a combination of group training, one‐on‐one sessions, material dissemination, and videos [S20].

### 3.5 Intervention target measures

There were large variations in target measures in all reviewed manuscripts. Many manuscripts used more than one target measure to evaluate their intervention. Of the reviewed articles, 26/37 measured knowledge [S2–S12, S15, S16, S18, S20, S21–S25, S29, S30, S32, S33, S35, S36], 17/37 measured health service utilization [S1, S2, S4, S5, S7, S12–S14, S17, S18, S21, S26, S27, S30, S33, S36, S37], 12/37 measured fever management skills [S3, S5, S6, S9, S10, S12, S17, S20–S23, S33], 8/37 measured medication dosing skills [S8, S15, S16, S18, S24, S25, S27, S31], 7/37 measured satisfaction [S1, S3, S4, S19, S23, S30, S33], 5/37 measured attitudes [S15, S16, S21, S24, S30], 3/37 measured influences [S15–S16, S24], 2/37 measured beliefs [S9, S11], 2/37 measured participants perceived confidence [S27, S35], 2/37 measured knowledge acquisition [S10, S19], and 1/37 measured behaviours [S6], motivations [S6], sales of medication [S27], anxiety [S28], number of prescriptions written [S31], cost per visit [S34], quality of documentation [S34], appropriateness of treatment [S34], and participants perceived innovation of the intervention [S35].

### 3.6 Intervention outcomes and tools used to measure them

Outcome tools used varied. These included pre–post questionnaires/surveys (20/37) [S2, S4, S6, S9–S11, S15, S16, S18, S19, S21–S25, S29, S30, S33, S35, S36], post‐only intervention questionnaire/surveys (2/37) [S12, S32], structured interviews (including telephone) (5/37) [S5, S9, S10, S17, S37], semi‐structured interviews (7/37) [S1, S3, S8, S23, S27, S28, S30], audits of healthcare facilities (including chart data, personnel, return visits, equipment/stock, and laboratory/diagnostic data) (13/37) [S5, S7, S12–S17, S20, S26, S31, S34, S37], and finally, one‐off measurement tools included the following: focus groups [S1], illness record books [S5], weekly performance review [S12], medical record checks [S18], medication sales data [S27], and patient treatment costs [S34].

Similarly, there was variation in when and how frequently the outcomes of each intervention were assessed. Measurements varied from being taken immediately following the intervention [S32] to scheduled monthly [S4] or yearly review [S22] (Appendix S3). Further, on top of a slew of different methods and structures used to measure educational interventions, duration of interventions ranged from short 3‐min videos [S3] to multiple yearlong training programmes [S1]. Additionally, while most studies used the measurement of knowledge to make an assessment of their interventions, there was no standardized fever knowledge tool used. Some studies used different combinations of target measures for the assessment of their interventions including sales data [S27], documentation quality [S34], or patient anxiety [S28] making correlation between interventions and assessed outcome increasingly difficult to establish.

The data contained in Table 1 were sorted firstly based on the type of intervention presented in the manuscript and then secondly sorted by the target audience of the interventions. Due to the large heterogeneity of the data, it is difficult to form many trends or assumptions regarding how effective the different types of interventions were. However, it can be extrapolated that video interventions were all aimed at parents/caregivers and provided positive improvements in the outcome measures “knowledge” and “satisfaction.” Peer‐to‐peer education interventions were all aimed at nurses and provided positive improvement in the outcome measure of “knowledge.” One‐on‐one session interventions were all aimed at parents/caregivers and provided positive improvements in the outcome measures “knowledge” and “health service utilization.” Group training session interventions aimed at parents/caregivers provided positive improvements in the outcome measures “knowledge” and “fever management skills.” Group training sessions interventions aimed at child health workers provided positive improvement in the outcome measure of “knowledge.”

**Table 1:** Simplified Table of data in Appendix S3 sorted by “Types of Intervention” and then sorted by “Who the training was aimed at”

Author	Type of intervention	Target of intervention	Positive outcome measures	Neutral/Negative outcome measures
Jeong YS, Kim JS. (2014) (S24)	Blended Learning	Nurses	K + D + A	I
Sanghavi D, et al. (2005) (S32)	Computerized Training	Caregiver	K	
Hart L, et al. (2016) (S19)	Computerized Training	Caregiver	KA + SA	
Pusic V, et al. (2012) (S29)	Computerized Training	Medical Students	K	
Schriger D, et al. (2000) (S34)	Computerized Training	Physicians	QD	APP + CPP
Chang L, et al. (2016) (S6)	Group Training Sessions	Caregiver	K + S + BE + M	
Chirdan O, et al. (2008) (S8)	Group Training Sessions	Caregiver	K	D
Huang M, et al. (1998) (S21)	Group Training Sessions	Caregiver	K + S + H + A	
Huang M, et al. (2002) (S22)	Group Training Sessions	Caregiver	K + S	
Fieldston E, et al. (2013) (S18)	Group Training Sessions	Caregiver	K + S	H
Wasunna B, et al. (2010) (S37)	Group Training Sessions	Child Health Workers	H	
Abbey M, et al. (2015) (S1)	Group Training Sessions	Child Health Workers	H + QS	
Eriksen J, et al. (2010) (S17)	Group Training Sessions	Community Wards	H + S	
De Vos‐Kerkhot E, et al. (2014) (S14)	Group Training Sessions	Healthcare staff		H
Statile A, et al. (2016) (S35)	Group Training Sessions	Medical Students	K + PC + II	
Cunningham A, et al. (2005) (S13)	Group Training Sessions	Primary Care Staff		H
Cropley L. (2004) (S12)	Group Training Sessions + One‐on‐one Sessions	Caregiver	K + S + H	
Chibwana A, et al. (2013) (S7)	Group Training Sessions + One‐on‐one Sessions	Healthcare staff	K + H	
Marsh V, et al. (1999) (S27)	Group Training Sessions + One‐on‐one Sessions	Shopkeepers	H + D + SM + PC	
Hu F, et al. (2016) (S20)	Group Training Sessions + One‐on‐one Sessions	Nurses + Caregiver	K + S	
Light P, et al. (2005) (S26)	Hotline Phone Calls	Caregiver	H	
Casey R, et al. (1984) (S5)	One‐on‐one Sessions	Caregiver	K + S + H	
Kelly L, et al. (1996) (S25)	One‐on‐one Sessions	Caregiver	K + D	
O'Neil‐Murphy K. (2001) (S28)	One‐on‐one Sessions	Caregiver	PA	
Sarrell M, Kahan E. (2003) (S33)	One‐on‐one Sessions	Caregiver	K + S + H + SA	
Steelman J, et al. (1999) (S36)	One‐on‐one Sessions	Caregiver	K + H	
Ruvinsky S, et al. (2013) (S31)	Peer‐to‐peer Education	Healthcare staff	ANT + D	
Considine J, Brennan D. (2007a) (S9)	Peer‐to‐peer Education	Nurses	K + S + B	
Considine J, Brennan D. (2007b) (S10)	Peer‐to‐peer Education	Nurses	K + S	KA
Edwards H, et al. (2007a) (S15)	Peer‐to‐peer Education	Nurses	K + D + I	A
Edwards H, et al. (2007b) (S16)	Peer‐to‐peer Education	Nurses	K + A + I	D
Considine J, Brennan D. (2006) (S11)	Peer‐to‐peer Education	Nurses	K + B	
Bloch S, Bloch A. (2013) (S3)	Video	Caregiver	K + S + SA	
Baker M, et al. (2009) (S2)	Video	Caregiver	K + H	
Ismail S, et al. (2016) (S23)	Video	Caregiver	K + S + SA	
Robinson J, et al. (1989) (S30)	Video	Caregiver	K + SA + H + A	
Broome M, et al. (2003) (S4)	Video + Written	Caregiver	K + SA + H	

Abbreviations: A: Attitudes; ANT: Antibiotic Prescriptions; APP: Appropriateness of Treatment; B: Beliefs; BE: Behaviour; CPP: Per‐patient Charge for treatment; D: Medication Dosing; H: Health Service Utilization; I: Influences; II: Intervention Innovation; K: Knowledge; KA: Knowledge Acquisition; M: Motivation; PA: Participant Anxiety; PC: Perceived Confidence; QD: Quality of Documentation; QS: Quality of Service; S: Fever Management Skills; SA: Satisfaction; SM: Sales of Medication.

Only 2/37 interventions (De Vos‐Kerkhot E, et al. 2014, Cunningham A, et al. 2005) provided no positive improvement in any outcome measures and only provided neutral results. Both of these manuscripts only targeted healthcare professionals, and both only looked at the outcome measure “health service utilization.”

Study bias was measured using either the STROBE checklist or Cochrane checklist for randomized control trials where applicable. Six manuscripts were evaluated as low risk of bias [S1, S2, S14, S19, S29, S30], eight were low‐to‐moderate risk [S3, S5, S9–S11, S18, S34, S35], nine had moderate risk [S6, S17, S22–S25, S31, S33, S36], seven had moderate‐to‐high risk [S4, S8, S15, S16, S26, S28, S37], and seven were classified with high risk [S7, S12, S13, S20–S21, S27, S32].

## 4 DISCUSSION

This review included 37 manuscripts that assessed the outcome of active educational interventions aimed at improving fever management in children. This is the first scoping review compiling all current “active” children's fever management interventions and aimed to compare the type of intervention used, who they targeted, what outcomes were measured, and what tools were used to measure outcomes. We therefore used these data to determine which intervention types were most effective in presenting fever management information. The results however highlighted that there was vast variation in how fever education has been delivered and assessed. In general, this review found that educational interventions improved knowledge of participant health professionals and caregivers, with video platforms being cited as a preferred medium for parents. Many interventions were created for small‐scale use and tailored for specific ethnic or regional groups leading to dispersed content based on individuals’ needs. The absence of an overarching generalized intervention makes it difficult to determine whether any specific single medium is appropriate for all target groups.

Three literature reviews on the topic of fever management in children and the success of current education methods have been published previously. Walsh et al. (2006) reviewed the literature concerning parental fever knowledge and beliefs in addition to educational interventions and aimed to understand caregiver's current attitudes, practices, and information seeking behaviours regarding children's fever and its management (Walsh & Edwards, 2006). They found that despite the reported success of many educational interventions, there is little that has changed in parent's fever knowledge, attitudes, and management practices, suggesting that future interventions should target behavioural change and focus on correcting inappropriate influences. Walsh also published a narrative review, which made no attempt to tabulate or compare the interventions studied (Walsh & Edwards, 2006).

Young et al.’s (2010) systematic review supported the notion that interventions based on behavioural change were necessary to improve fever management outcomes in parents. They concluded that multidimensional interventions using mixed methods and repletion/reinforcement were most effective, but there were few studies to compare (*N* = 10) to confirm these findings. This review also stated that healthcare providers have difficulty disseminating fever information to parents; however, the review did not focus on healthcare providers.

Monsma et al. (2015) reviewed the factors that should be considered when designing an educational intervention aimed at improving caregivers fever management with focus on low health literacy participants, recommending that one to one, structured, multidimensional, and reinforced over time sessions were the most effective educational interventions. They also suggested that culturally sensitive interventions catered to the target audience would maximize translation of best evidence into practice. Monsma's review therefore had a narrower focus than our review.

Therefore, our review, while comparable to the aforementioned published literature, has expanded on their work to include a broader focus on interventions targeted towards all groups as “fever phobia” is not just a parental issue (El‐Radhi, 2008). We propose that educational interventions should focus on both caregivers and health professionals as to date focusing on only one group has not seemed to dramatically change either groups’ attitudes towards “fever phobia.”

The most common form of intervention used in the studies included in our review was group training sessions [S1, S6–S8, S12–S14, S17, S18, S20–S22, S27, S35, S37] which included lectures, discussions, tutorials, or a combination of these at any point in the intervention. This provided the highest percentage of intervention type perhaps as these educational interventions require less resources to produce and could be perceived as easier to conduct than their counterparts. Further, this face‐to‐face form of teaching allows for demonstration. According to a meta‐analysis by Theis (1995), demonstration is seen as the teaching strategy to have the greatest influence on effect size in an intervention, followed by computer simulated and audio and visual with verbal instruction seen as the least effective strategy. It was also shown that using multiple teaching methods is a good strategy to allow the highest effect size of the intervention compared with standard care or control groups (Friedman, Cosby, Boyko, Hatton‐Bauer, & Turnbull, 2011; Theis & Johnson, 1995). However, it is to be noted that Monsma et al. (2015) stated that interventions, which are largely structured, generally result in better effect size outcomes regardless of the type of intervention used. Of the interventions that specifically listed demonstrations as part of the intervention [S5, S18, S22], they all showed significant improvement in fever management skills [S5, S22], health service utilization [S5, S18], and knowledge [S5, S18, S22].

Abbey [S1] showed that their video intervention was more easily understood and recollected compared with audio and group talks/seminars [S1] and the study by Robinson [S30] highlighted that participants wanted more audio/visual health programmes [S30]. In both these cases, the satisfaction of the participants receiving video intervention was significantly higher than the control group counterparts. Education through video format has had mixed results in other fields (Friedman et al., 2011). Educational experts will often suggest that a blended approach to learning; that is, a mix of video/online media plus face‐to‐face is more effective that either method alone (Means, Toyama, Murphy, & Bakia, 2013). However, video/online media do have the advantage of gaining further reach as the participant and teacher do not need to be present in the same classroom. Further research in using online or video education that is soundly based on pedagogical principals and is engaging for the learner needs attention, specifically in the area of caregiver fever management where all parents will find themselves needing knowledge to manage a fever at some stage.

Parents and caregivers were by far the largest percentage target of fever management interventions. All but two of the interventions [S26, S28] aimed at parents focused on the outcome measurement of knowledge. It is known that knowledge underpins competency (Miller, 1990) and this is an important outcome to measure, due to the conflicting information that caregivers receive regarding fever management. However, other studies have also highlighted that education should focus on skills development—the higher levels of Millar's competency pyramid (Miller, 1990) as the “Knows” and “Knows how” region are mainly intermediary markers and not true reflections of lasting change in practice. In particular, the functional health literacy of caregivers and their accuracy in measuring doses have been shown to be exceptionally poor (Emmerton et al., 2014; Hietbrink, Bakshi, & Moles, 2014; Parker & Gazmararian, 2003). In fact, medication dosing was only measured in 8/37 of manuscripts, and of those reviewed, only two articles by Kelly [S25] and Marsh [S27] used an observed method with the remainder focusing on parent's intention to treat or measuring clinicians’ adequate use of medications.

Many of the studies in fact did not seem to have an assessment measure constructively aligned (Biggs, 2003) with the educational intervention objectives. For example, the study by Huang [S21] aimed to improve anticipated measures parents took when their child had a febrile convulsion, yet the outcomes were measured through self‐reported survey; hence, we have no real knowledge of effectiveness. These higher order outcomes need their measurement tool to be constructively aligned to guarantee that the skills taught have a long‐lasting effect and consolidation. Recent articles by Hietbrink (2014) and Emmerton (2014) observed that the proportions of parent with skills to dose their child accurately on weight were between 30%–33%, highlighting that parents continue to inappropriately handle medication dosing for children with fever. Therefore, it is imperative that future educational interventions targeting medication dosing skills should be aimed to assess and improve outcomes at higher levels of Miller's pyramid and should be evaluated through observation, rather than through self‐reported survey.

In this review, all studies were included regardless of their bias rating due to low numbers of studies found. As stated, most studies showed positive effects of the educational interventions. It is possible that publication bias may have contributed and caused interventions with neutral or negative results to not be published. Study bias ranged from the very high to very low risks; however, most studies provided Level II (Newhouse, Dearholt, Poe, Pugh, & White, 2005) evidence through quasi‐experimental and pre‐ to post‐test studies. Many of these studies suffered from issues in bias including small sample sizes and loss of data due to attrition. Of the RCTs, none fully used all blinding processes or described in detail the processes of randomization or concealment of results. Many also suffered from poor generalizability, leading to a low credibility of results and difficulty making conclusions on the long‐term and large‐scale usability of these children's fever educational interventions. On the other hand, on face value, if educational interventions are working for the small groups of research participants in the included studies, it would seem evident that further roll‐out of these interventions as part of a public health campaign has been lacking.

This scoping review had the purpose to compile a comprehensive list of articles and manuscripts relevant to fever management interventions aimed at both healthcare providers and caregivers. With this, we believe that the findings from this review can form the foundations to aid researchers and educators in developing new educational fever management interventions for future studies. However, our review is not without its own limitations including the exclusion of non‐English studies and studies published prior to January 1980; hence, some studies may be missing. However, our search strategy was aligned with the release of Schmitt's (1980) identification of the term “fever phobia” and hoped to capture most literature written after the publication of this idea. Finally, the STROBE checklist was designed originally as a measure of quality for authors rather than a bias assessment tool; hence, its use for bias assessment may lack validity.

## 5 CONCLUSION

This review compared educational mediums, who they were aimed at, what targets were measured, and what tools were used to measure outcomes of fever educational interventions. Mostly positive data pose a challenge in determining education interventions “effectiveness” and if any long‐lasting outcomes on participant's knowledge and behaviours are affected. The absence of wide‐scale interventions of any medium makes it difficult to determine whether any of these interventions have had impact on reducing “fever phobia.” The lack of standardized approaches to fever education targeted at both caregivers and healthcare providers, and the assessment of their outcomes makes it difficult to draw any firm conclusions on the best educational tools in this field.

## CONFLICTS OF INTEREST

The authors have no conflicts of interest to declare.

## AUTHORS CONTRIBUTIONS

D.A., T.C., R.M.: substantial contributions to conception and design, or acquisition of data, or analysis and interpretation of data; drafting the manuscript or revising it critically for important intellectual content; final approval of the version to be published; and agreed to be accountable for all aspects of the work in ensuring that questions related to the accuracy or integrity of any part of the work are appropriately investigated and resolved. Each author should have participated sufficiently in the work to take public responsibility for appropriate portions of the content.

## DATA AVAILABILITY

All data generated or analysed during this study are included in this published article [and its Supplementary information files].

## References

[nop2294-bib-0001] Abdullah, M. A. , Ashong, E. F. , Al Habib, S. A. , Karrar, Z. A. , & Al Jishi, N. M. (1987). Fever in children: Diagnosis and management by nurses, medical students, doctors and parents. Annals of Tropical Paediatrics, 7(3), 194–199. 2445269 10.1080/02724936.1987.11748506

[nop2294-bib-0002] Baker, M. D. , Monroe, K. W. , King, W. D. , Sorrentino, A. , & Glaeser, P. W. (2009). Effectiveness of fever education in a pediatric emergency department. Pediatric Emergency Care, 25(9), 565–568. 10.1097/PEC.0b013e3181b4f64e 19755888

[nop2294-bib-0003] Berry, A. , Brousseau, D. , Brotanek, J. M. , Tomany‐Korman, S. , & Flores, G. (2008). Why do parents bring children to the emergency department for nonurgent conditions? A Qualitative Study. Ambulatory Pediatrics, 8(6), 360–367. 10.1016/j.ambp.2008.07.001 19084785

[nop2294-bib-0004] Biggs, J. (2003). Aligning teaching and assessing to course objectives (Vol. 2).

[nop2294-bib-0005] Blumenthal, I. (1998). What parents think of fever. Family Practice, 15(6), 513–518. 10078789 10.1093/fampra/15.6.513

[nop2294-bib-0006] Chiappini, E. , D'Elios, S. , Mazzantini, R. , Becherucci, P. , Pierattelli, M. , Galli, L. , & de Martino, M. (2013). Adherence among Italian paediatricians to the Italian guidelines for the management of fever in children: A cross sectional survey. BMC Pediatrics, 13, 210 10.1186/1471-2431-13-210 24350822 PMC3878332

[nop2294-bib-0007] Considine, J. , & Brennan, D. (2007). Effect of an evidence‐based education programme on ED discharge advice for febrile children. Journal of Clinical Nursing, 16(9), 1687‐1694. 10.1111/j.1365-2702.2006.01716.x 17727587

[nop2294-bib-0008] Crocetti, M. , Moghbeli, N. , & Serwint, J. (2001). Fever phobia revisited: Have parental misconceptions about fever changed in 20 years? Pediatrics, 107(6), 1241–1246. 11389237 10.1542/peds.107.6.1241

[nop2294-bib-0009] Demir, F. , & Sekreter, O. (2012). Knowledge, attitudes and misconceptions of primary care physicians regarding fever in children: A cross sectional study. Italian Journal of Pediatrics, 38(1), 40 10.1186/1824-7288-38-40 22950655 PMC3481471

[nop2294-bib-0010] El‐Radhi, A. S. M. (2008). Why is the evidence not affecting the practice of fever management? Archives of Disease in Childhood, 93(11), 918–920. 10.1136/adc.2008.139949 18562453

[nop2294-bib-0011] El‐Radhi, A. S. M. (2012). Fever management: Evidence vs current practice. World Journal of Clinical Pediatrics, 1(4), 29–33. 10.5409/wjcp.v1.i4.29 25254165 PMC4145646

[nop2294-bib-0012] Emmerton, L. , Chaw, X. Y. , Kelly, F. , Kairuz, T. , Marriott, J. , Wheeler, A. , & Moles, R. (2014). Management of children's fever by parents and caregivers: Practical measurement of functional health literacy. Journal of Child Health Care, 18(4), 302–313. 10.1177/1367493513496663 23908369

[nop2294-bib-0013] Friedman, A. J. , Cosby, R. , Boyko, S. , Hatton‐Bauer, J. , & Turnbull, G. (2011). Effective teaching strategies and methods of delivery for patient education: A systematic review and practice guideline recommendations. Journal of Cancer Education, 26(1), 12–21. 10.1007/s13187-010-0183-x 21161465

[nop2294-bib-0014] Grimshaw, J. M. , Thomas, R. E. , MacLennan, G. , Fraser, C. , Ramsay, C. R. , Vale, L. , … Donaldson, C. (2004). Effectiveness and efficiency of guideline dissemination and implementation strategies. Health Technology Assessment, 8(6), iii–iv, 1–72. 10.3310/hta8060 14960256

[nop2294-bib-0015] Hewson, P. (2000). Paracetamol: Overused in childhood fever. Australian Prescriber, 23(3), 2 10.18773/austprescr.2000.063

[nop2294-bib-0016] Hietbrink, E. , Bakshi, R. , & Moles, R. J. (2014). Australian caregivers' management of childhood ailments. The International Journal of Pharmacy Practice, 22(3), 205–215. 10.1111/ijpp.12067 24033690

[nop2294-bib-0017] Higgins, J. P. , Altman, D. G. , Gotzsche, P. C. , Juni, P. , Moher, D. , & Oxman, A. D. , … Cochrane Statistical Methods Group (2011). The Cochrane Collaboration's tool for assessing risk of bias in randomised trials. BMJ, 343, d5928 10.1136/bmj.d5928 22008217 PMC3196245

[nop2294-bib-0018] Kluger, M. (1979). Fever: Its biology, evolution and function. Princeton, NJ: Princeton University Press.

[nop2294-bib-0019] May, A. , & Bauchner, H. (1992). Fever phobia: The pediatrician's contribution. Pediatrics, 90(6), 851–854. 1437424

[nop2294-bib-0020] Means, B. , Toyama, Y. , Murphy, R. , & Bakia, M. (2013). The effectiveness of online and blended learning: A meta‐analysis of the empirical literature. Teachers College Record, 115(3), 713–47.

[nop2294-bib-0021] Miller, G. E. (1990). The assessment of clinical skills/competence/performance. Academic Medicine, 65(9 Suppl), S63–67. 2400509 10.1097/00001888-199009000-00045

[nop2294-bib-0022] Moher, D. , Liberati, A. , Tetzlaff, J. , & Altman, D. G. (2009). Preferred reporting items for systematic reviews and meta‐analyses: The PRISMA statement. PLoS Medicine, 6(7), 10.1136/bmj.b2535 PMC2707599 19621072

[nop2294-bib-0023] Monsma, J. , Richerson, J. , & Sloand, E. (2015). Empowering parents for evidence‐based fever management: An integrative review. Journal of the American Association of Nurse Practitioners, 27(4), 222–229. 10.1002/2327-6924.12152 25066313

[nop2294-bib-0024] National Collaborating Centre for Women's and Children's Health (UK) (2013). NICE guideline: Feverish illness in children ‐ Assessment and initial management in children younger than 5 years, Vol. C160 London: National Institute for Health and Care Excellence.

[nop2294-bib-0025] Newhouse, R. , Dearholt, S. , Poe, S. , Pugh, L. C. , & White, K. M. (2005). Evidence‐based practice: A practical approach to implementation. Journal of Nursing Administration, 35(1), 35–40. 15647668 10.1097/00005110-200501000-00013

[nop2294-bib-0026] NSWHealth , (2010). Infants and children: acute management of fever / NSW Health. North Sydney, N.S.W: NSW Dept. of Health.

[nop2294-bib-0027] O'Neill‐Murphy, K. , Liebman, M. , & Barnsteiner, J. H. (2001). Fever education: Does it reduce parent fever anxiety? Pediatric Emergency Care, 17(1), 47–51. 11265909 10.1097/00006565-200102000-00014

[nop2294-bib-0028] Parker, R. M. , & Gazmararian, J. A. (2003). Health literacy: Essential for health communication. Journal of Health Communication, 8(1 Suppl), 116–118. 10.1080/713851963 14692576

[nop2294-bib-0029] Raffaeli, G. , Orenti, A. , Gambino, M. , Peves Rios, W. , Bosis, S. , Bianchini, S. , Tagliabue, C. , & Esposito, S. (2016). Fever and pain management in childhood: healthcare providers' and parents' adherence to current recommendations. International Journal of Environmental Research and Public Health, 13(5), 499 10.3390/ijerph13050499 PMC4881124 27187436

[nop2294-bib-0030] Richardson, M. , & Purssell, E. (2015). Who’s afraid of fever? Archives of Disease in Childhood, 100(9), 818–820. 10.1136/archdischild-2014-307483 25977564

[nop2294-bib-0031] SAHealth . (2013). Management of Fever without Focus in Children (excluding neonates) Clinical Guideline. S.A.: Government of South Australia.

[nop2294-bib-0032] Schmitt, B. (1980). Fever phobia. Misconceptions of parents about fevers. American Journal of Diseases of Children, 134(2), 176–181. 7352443

[nop2294-bib-0033] Shakeel, S. , Iffat, W. , & Qamar, A. (2014). Physicians’ apprehensions in managing a febrile child. Asian Journal of Pharmaceutical and CLinical Research, 7(5), 173–177.

[nop2294-bib-0034] So, E. , & Moles, R. (2014). Caregivers’ Management of Childhood Fever and their Rationale for their Practice. Dissertation. School of Pharmacy. University of Sydney. Sydney.

[nop2294-bib-0035] Soszynski, D. (2003). The pathogenesis and the adaptive value of fever. Postȩpy Higieny I Medycyny Doświadczalnej, 57(5), 531–554. 14737969

[nop2294-bib-0036] Theis, S. L. , & Johnson, J. H. (1995). Strategies for teaching patients: A meta‐analysis. Clinical Nurse Specialist, 9(2), 100–105, 120. 7600475 10.1097/00002800-199503000-00010

[nop2294-bib-0037] von Elm, E. , Altman, D. G. , Egger, M. , Pocock, S. J. , Gotzsche, P. C. , & Vandenbroucke, J. P. (2007). The Strengthening the Reporting of Observational Studies in Epidemiology (STROBE) statement: Guidelines for reporting observational studies. Annals of Internal Medicine, 147(8), 573–577. 17938396 10.7326/0003-4819-147-8-200710160-00010

[nop2294-bib-0038] Walsh, A. , & Edwards, H. (2006). Management of childhood fever by parents: Literature review. Journal of Advanced Nursing, 54(2), 217–227. 10.1111/j.1365-2648.2006.03802.x 16553708

[nop2294-bib-0039] Walsh, A. , Edwards, H. , & Fraser, J. (2008). Parents' childhood fever management: Community survey and instrument development. Journal of Advanced Nursing, 63(4), 376–388. 18727765 10.1111/j.1365-2648.2008.04721.x

[nop2294-bib-0040] Young, M. , Watts, R. , & Wilson, S. (2010). The effectiveness of educational strategies in improving parental/caregiver management of fever in their child: A systematic review. JBI Database of Systematic Reviews and Implementation Reports, 8(21), 826–868. 10.11124/01938924-201008210-00001 27820024

[nop2294-bib-0041] Zyoud, S. H. , Al‐Jabi, S. W. , Sweileh, W. M. , Nabulsi, M. M. , Tubaila, M. F. , Awang, R. , & Sawalha, A. F. (2013). Beliefs and practices regarding childhood fever among parents: A cross‐sectional study from Palestine. BMC Pediatrics, 13, 66 10.1186/1471-2431-13-66 23622106 PMC3641948

