# Supplementary material for: Educational interventions on fever management in children: A scoping review
Source: Nurs Open. 2019 May 1;6(3):713–21. doi: 10.1002/nop2.294 (PMC6650695; doi:10.1002/nop2.294)

**Appendix 1**

The following keywords and Medical Subject Headings (MeSH) were initially searched in MEDLINE utilizing the ‘explode’ function:

(‘fever’ [MeSH] OR ‘febrile’ [MeSH] OR ‘temperature’ [MeSH] OR high temperature [keyword])

AND

(‘child’ [MeSH] OR ‘children’ [keyword] OR ‘infant’ [MeSH] OR ‘pediatric’ [MeSH]

AND

‘health education’ [MeSH] OR ‘education’ [MeSH] OR ‘nursing education’ [MeSH] OR ‘parent education’ [keyword] OR ‘caregiver education’ [keyword] OR ‘parent’ [MeSH] OR ‘caregiver(s)’ [MeSH] OR ‘simulation’ {MeSH] OR ‘patient education as a topic’ [MeSH] OR ‘interactive learning’ [MeSH] OR ‘simulation training’ [keyword] OR ‘training’ [keyword] OR ‘intervention’ [keyword] OR ‘health intervention’ [MeSH] OR ‘health promotion’ [MeSH] OR ‘pilot project(s)’ [MeSH]).

These search terms were then mapped to equivalent subject headings and exploded in EMBASE, CINHAL, PsycINFO and PubMED and keyword searches were used in IPA

**Appendix 2**

Sample search strategy utilised in OVID Medline


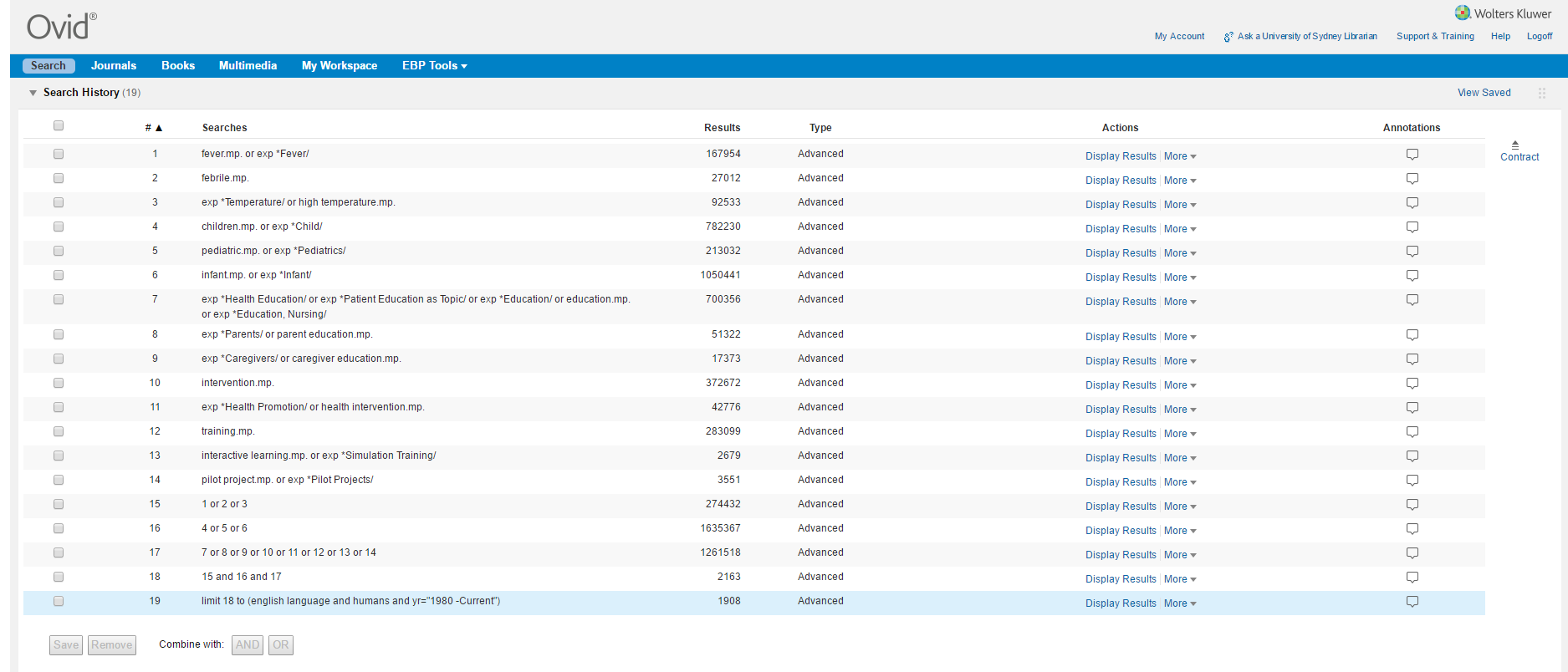


Sample search strategy utilised in CINHAL


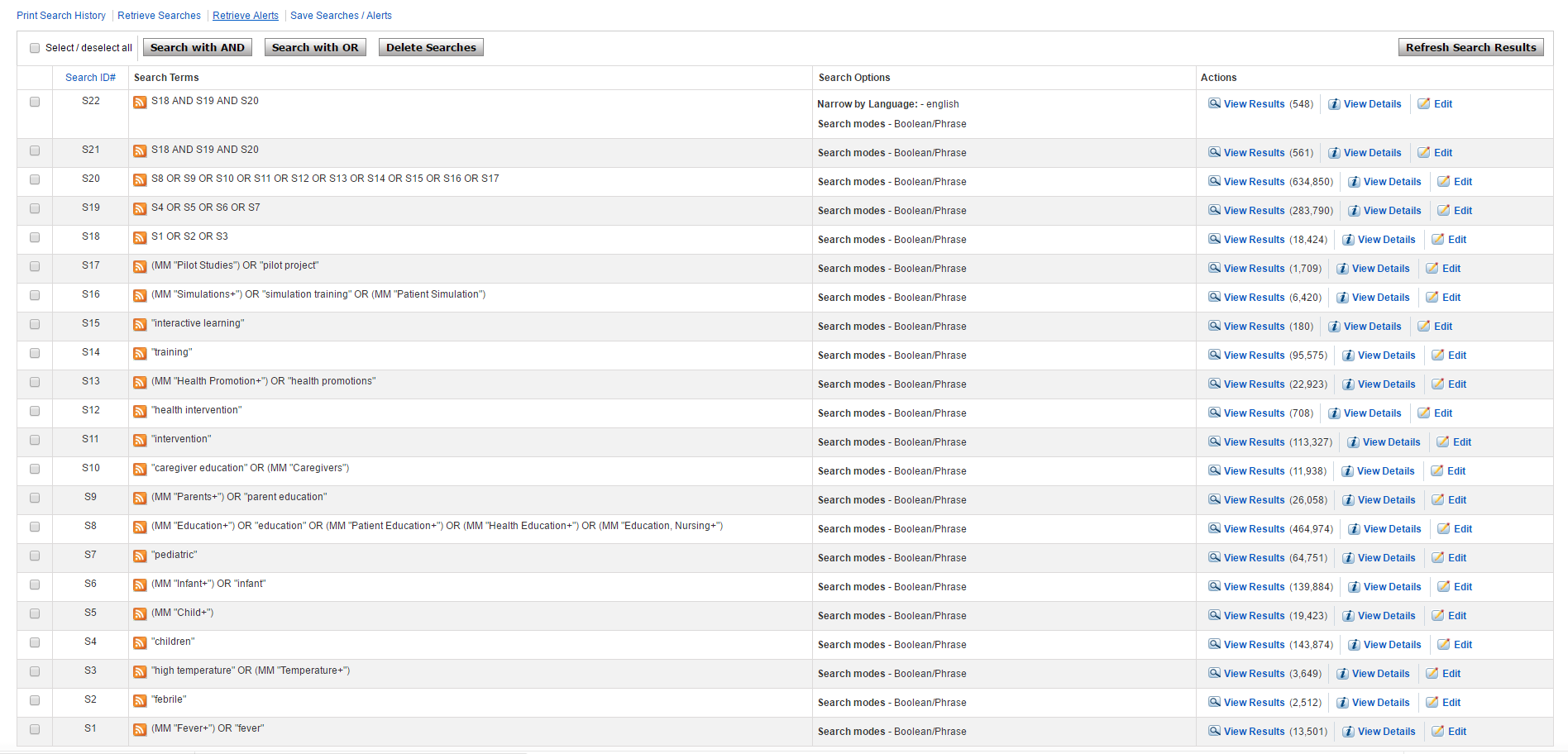

Supplement: Supplementary file 1 [file NOP2-6-713-s001.docx]
