# Supplementary material for: Educational interventions on fever management in children: A scoping review
Source: Nurs Open. 2019 May 1;6(3):713–21. doi: 10.1002/nop2.294 (PMC6650695; doi:10.1002/nop2.294)
Supplement: Supplementary file 2 [file NOP2-6-713-s002.docx]

**Appendix 3: Summary of Articles reviewed containing education intervention for fever management**

| **Author, Year and Location** | **Study Design** | **Aim of Study** | **Setting of the Paper** | **Participants (who was the study aimed at?)** | **Sample Size** | **Intervention** | **How was it measured?** | **Study Measures** | **Findings/Results** | **Bias Potential** |
| --- | --- | --- | --- | --- | --- | --- | --- | --- | --- | --- |
| Abbey M, et.al  (2015) (S1)  **Ghana** | Cross sectional survey | To assess the community utilization, perceptions and related factors of community health worker services for fever in children under 5 in the Dangeme West district of Ghana | Community/ Public health facilities | Child Health Workers (CHW) in the Dangme district were trained to disseminate information to caregivers of children under 5 | **660 CHW were trained. (12 focus groups were done involving 84 caregivers.**  **Data were collected from 562 caregivers through an interview)** | **Group Training Sessions:**  CHW were selected by their communities and trained by professional health staff  3-day training sessions using UNICEF and WHO guidelines  A complementary communications program was implemented in all communities. The messages were disseminated through oral presentations, audio tapes, mobile van and a locally-produced video to enhance awareness and utilization of CHW services in the communities. Key messages delivered covered the rationale for the trial, the availability of trained CHWs in the communities for prompt and appropriate management of fevers in children under 5 and the importance of adhering to treatment and referral. | 12 focus groups with 84 caregivers were done to capture variable experiences and in-depth discussions of perceptions of the intervention  An interview was undertaken with all caregivers which participated in the survey (using a questionnaire with both closed and open ended questions (done whether or not were aware of or exposed to the CHW’s) | Health service utilization  Quality of service (satisfaction) | - Overall 59.4% (334/562) used CHW services for fever during the 30-month intervention period (Oct 2009). This was a steady increase from 26.4% (169/640) in April 2008. - Of the 59.4% (334/562) caregivers who utilized CHW services, 45.0% (253/562) perceived the quality of the services received as ‘good’, while 13.7% (77/562) described the service as ‘excellent’. The rest 0.71% (4/562) assessed the services as fair. - Regression showed that caregivers who were exposed to the intervention were 4 times more likely to use services of the CHW compared to the control (OR= 3.9, p <0.001) - Caregiver cited benefits from the accessed medicine in treating fever resulting in a reduction in reported fever cases among children in the community compared to the time before the intervention. Most also stated there was an elimination of costs due to ease of access to CHW’s - Of the 56.1% who either saw or heard the communications, 55.8% (314/562) reported that the messages delivered were appropriate, understandable and acceptable - 58.9% (331/562), indicated that they would recommend the CHW who attended to them to other caregivers. - 56.1% (315/562) of caregivers indicated personal awareness of the program through participation in campaign activities. A majority, 93.1% (523/562) of caregivers reported that they had knowledge about CHWs in their communities. - Of all materials disseminated, the video was the most easily recollected and understood (compared to the audio and the talk | Study size not justified  Data measurement resource not well explained  **LOW RISK** |
| Baker M, et.al  (2009) (S2)  **United States** | Prospective Randomised Cohort Study | To examine if a brief video about the home management of fever presented to parents during an ED visit for minor febrile illness would affect unnecessary return to the ED | Urban paediatric Emergency Departments (ED) | Parents / caregivers with a child between 3 – 36 months old | **280 parents/ caregivers (140 received control video and 140 received the intervention video)** | **Video:**  Intervention was an 11-minute video on the home management of fever (Control also got a video 8 minutes on automobile safety) | A pre-test and post-test survey was given  Both surveys were identical and contained 7 items (regarding general fever knowledge and treatments)  Subjects were followed up for return visits to the ED (medical necessity was determined by 3 reviewers) | Knowledge  Health service utilization | - Intervention group had 54% reduction in respondents who reported fever was dangerous by itself (p < 0.001). - Intervention group had 28% reduction in parents who reported to wake the child to treat fever (p < 0.0001) - Intervention group displayed a 30% improvement in respondents who could identify that aspirin was an inappropriate choice (p < 0.0001) - Pre-test and post-test data showed similar results for the control group - Pre-test data for both control and intervention were nearly identical - No difference was seen in the rate of return the ED with febrile illness between the intervention (56 patients and 81 visits) and control (49 patients and 81 visits) groups (p = 0.46) - There was an improvement in the amount of medically necessary visits in the intervention group (37%) over the control (25.9%) however this was not significant (p = 0.07) | Follow-up and missing data not specified  Participants obtained through convenience sampling  **LOW RISK** |
| Bloch S, Bloch A  (2013) (S3)  **United States** | RCT | To compare if video discharge instructions will improve caregiver’s comprehension of their child’s medical condition, treatment and follow-up and improve caregiver’s satisfaction (over written discharge information) | One academic paediatric ED Georgia, USA | Parents and caregivers of children aged 1 month to 18 years old | **436 parents/ caregivers**  **(220 received written and 216 received video)**  89 caregivers in each group received Fever information | **Video:**  Intervention was a 3-minute video discharge instruction (comparable to the standard written instructions) regarding fever, vomiting, diarrhoea or asthma | Informal interview asked for demographic information  5 verbal questions and a 20-item questionnaire (diagnosis, treatment and follow-up) given after the intervention  Informal interview asked for patient satisfaction  Then post discharge questionaries were given 2-5 days after (same as above) | Knowledge  Fever Management Skills  Satisfaction | - In all instances the video discharge group scored a higher average score than the written group (in all diagnosis and both ED and 2-5 days after) - For fever, the average scores were found to be significant for the video group over the written group at both ED (Written = 9.1 Video = 13.1 p < 0.0001) and 2-5 days’ follow-up (Written = 7.5 Video = 11.5 p < 0.0001) - Those receiving video discharge information rated their satisfaction more positively than written instructions, however this was not found to be statistically significant - However, satisfaction was significantly higher at the 2-5-day follow-up over the initial ED (p < 0.05) as caregivers valued the take home information. | Blinding of participants not specified  No Blinding of assessors (used a strict format interview)  20-25% attrition  **LOW-MOD RISK** |
| Broome M, et.al  (2003) (S4)  **United States** | RCT | To examine the effectiveness of a structured education program to increase parents or grandparents immediate and long term retention of knowledge, confidence, and satisfaction in assessing a child’s fever. | 6 sites including 2 private practices, 3 children’s hospitals and 1 academic medical centre | Parents and grandparents of children from 3-80 months’ old | **216 parents/ grandparents**  **(189 followed up at 3 months, 145 followed up at 6 months)**  **Split into 3 groups:**  **-Control**  **-CALM1 (given intervention before seeing HCP)**  **-CALM2 (given intervention after leaving the clinic)** | **Video and Written information**:  Intervention was the CALM brochure (developed by AAP guidelines) followed by a video reinforcing the information. | 2 questionnaires (1 demographic and the other the fever management questionnaire FMQ)  Follow-up 15-minute interview repeating the FMQ at 24h, 72h, 1 month, 3months and 6 months  Questions 1-14 are MCQ assessing fever understanding  Questions 15-18 are a 10-point scale on satisfaction with HCP  Questions 19-23 request how often they attend a HCP or get advice  Question24-36 pertains the CALM intervention and how useful it was | Knowledge  Satisfaction  Health service utilization | - Significant increase in total knowledge scores across all 3 groups from baseline through to 6 months (p < 0.01) - Both CALM groups had significantly higher knowledge than the control group at all data collection times (p < 0.03) - By 6 months only the CALM2 group which had been given individualized instructions in addition to the brochure and video had significantly higher score than the control group. - Satisfaction of both the control and CALM groups increased over time and there were no significant differences. Both CALM groups were higher than the control groups (p = 0.08) - Report levels of confidence in calling their HCP increased significantly (p < 0.05) from baseline. However, the CALM groups were not different from each other. - Overall parents using both CALM approaches were positive of their approach. With scores ranging from 61% to 98% of each cohort responding positively. | Locations use not randomized  Randomization method not specified  Assessor blinding not specified  While its only61-98%, all groups were 90% average except one group in CALM2 which only scores 65% average satisfaction  **MOD- HIGH RISK** |
| Casey R, et.al  (1984) (S5)  **United States** | Prospective cohort study | Aims were to:  1: dispel parent’s misconceptions about fever and teach them to initiate proper care of their febrile child  2: reduce the misuse of anti-pyretics  3: provide parents with a list of serious signs that warrant medical attention  4: decrease the inappropriate use of HCP for minor febrile illness | Private group practice at the Children’s Hospital of Philadelphia | Parents / Caregivers of children aged 6 months to 4 years | **106 parents of 108 children (53 in intervention and 53 in the control groups)**  There were 54 children in each group | **One-on-One sessions:**  The intervention was a standardized interview lasting 20 minutes at the children’s hospital which the management of fever was discussed, demonstrated and practiced. | Parents were interviewed (structured 15 items on knowledge and management of fever) by research assistants to get baseline data.  This was followed by a telephone interview 2 months and 4 months repeating the structured interview  The parents were also asked to fill in an ‘illness record’ of all actions taken when I child has illness over a 2-month period (from when the intervention ended to both telephone interviews)  Physician contact was also measured as well as chart audits | Knowledge  Fever Management Skills  Health service utilization | - Despite high education, many of the parents were misinformed about aspect of the management of fever. - Both groups showed similar increases in knowledge (not significant) as indicated by 2^nd^ interview responses - The intervention group had significant changes in their patterns of fever management. Total inappropriate visits to Physicians was significantly lower in the intervention group over the control (9.5% vs 38.5%) (p = 0.05). Also, similar trends were seen in inappropriate telephone calls to physicians (26.1% vs 56.3%) (p = 0.02) - The amount of episodes anti-pyretics were inappropriately used in fever significantly improved in the intervention group over the control (12.7% vs 42.6%) ( p < 0.005) - Interviews to assess parental knowledge of fever management at the end of the study were obtained for 94/106 parents. Additionally, illness records were returned for 92/108 children at 2 months and 81/108 children at 4 months | No attempt to control bias  Non-participation reasons unspecified  **LOW-MOD RISK** |
| Chang L, et.al  (2016) (S6)  **Taiwan** | Quasi-experimental pre-post study | Aimed to assess the long-term effect of simulation based education on parental fever management, motivation, behavioural skills and management behaviours. | Location of intervention not specified  (community location implied) | Parents of kindergarten age children (3 months to 5 years), not currently suffering from fever or disease | **160 children and their caregivers:**  **80 control group and 80 intervention group** | **Group training sessions:**  Parents of the intervention group attended a 30-minute fever care simulation session which covered:  1 – knowledge of fever  2 – assessing symptoms for which a single antipyretic was not required  3 – assessing symptoms for which a single antipyretic was required  4 – deciding when to seek medical advice  5 – discussion  After this parents of both intervention and control group received a fever education brochure and were explained key points and content. | Participants were given a survey pre-intervention, post intervention and then follow-up was undertaken over the phone at 6 and 12 months. Questionnaire was the same at each timepoint.  The questionnaire was 24 items and split:  -6 items on fever information  -7 items where regarding motivations  -6 items were regarding behaviour  -5 items where regarding fever management behaviours | Knowledge  Fever Management Skills  Motivation  Behaviours | - 1 participant dropped out of both the control and experimental group leaving the final tally as 79 in both groups respectively - There was no significant difference between control and experimental groups in the pre-test surveys, however there are significant differences (p < 0.001) in the post test, 6 month and 12 month surveys. - Fever information (I), motivation (M), behaviour (B) and management behaviours (Mb) levels of improvement in the experimental group where all significantly greater than that of the control group at all time points (p < 0.01) - Mean score difference between post and pre-test in the experimental group was:1.85 (I), 4.56 (M), and 4.40 (B) (at 1 day). All values where statistically significant at the 6 month and 12-month post tests - Mean score difference in the control group was statistically significant for the first day pre-test, however scored dropped quickly after and where not significantly different for the 6-month and 12-month tests. | Study design not stated  Not specified where the intervention took place  Not specified who was in charge of the simulation scenario  Initial time study started not specified  Confusing use of abbreviations – makes information hard to decipher  **MOD RISK** |
| Chibwana A, et.al  (2013) (S7)  **Malawi** | Pre-post study | Aimed to assess current practice for the management of paediatric febrile events against the evidence based best practice recommendations and thereby improve child health outcomes in Limbe Health Centre Malawi. | Limbe health Centre | Facility staff comprising clinicians, nurses and Health Surveillance Assistants’ (HSAs) | **20 children were followed from initial assessment and 20 more after the interventions**  **16 facility staff comprising of 4 clinicians, 5 nurses and 7 HSAs were taught basics in triaging by the study clinicians** | **Group training, one-on-one sessions and material dissemination:**  After baseline audits, a meeting was called with the facility staff (clinicians, nurses and health surveillance assistants) and District Medical Officer (DMO) representative to present the audit results and discuss obstacles or barriers preventing professionals from implementing evidence based practice  Implemented strategies including: Supply thermometers and anti-pyretics, education on how to complete a full assessment, distribution of standardized tools for assessing the child and posters on management  Knowledge was improved with, group training sessions (for teaching importance of triage) and one-on-one education (study clinicians gave feedback to faculty staff on management skills) and discussion with DMO’s  Following the implementation of the education strategies a follow up audit was conducted using the same audit criteria and sample size as baseline audit  All undertaken by the project team and DMO’s | Baseline audit was done at Limbe Health Centre during July 2013 for three days.  The 4 audit criteria used:  1. A full assessment of the child carried out to predict risk of SBI  2. Anti-pyretics are administered as clinically indicated  3. During triage the temperature of child is taken  4. Parents /carers are given info on fever management prior to discharge from the OPD department | Knowledge  Health service utilization | - Poor performance was evident from the baseline audit, in all of the criteria, meaning that there were serious gaps between current practice and evidence based practice. - Similarly, 90% (n=20) of the children had temperature taken on triage compared to 45% at baseline. - Antipyretics are administered as clinically indicated increased from 0% to 100% after the intervention - Temperature of the child is taken to assess for presence of fever during triage increased from 45% baseline to 100% compliance post interventions - Parents/carers given information on fever management prior to discharge from the Outpatient Department also increased from 0% compliance to 100% - Parents/carers were not discouraged to use physical cooling methods such as tepid sponging to reduce fever at home, because Malawi promotes physical cooling methods in the guidelines.   This set of results was discussed to be a reflection on the effectiveness of the one-on-one teaching method, and group teaching sessions | Difference cohort used for pre/post data  No eligibility criteria  Sample size not justified (and is small)  **HIGH RISK** |
| Chirdan O, et.al  (2008) (S8)  **Nigeria** | Pre-Post study | To explore the responsiveness of mothers to information on home management and proper treatment seeking behaviour about childhood fevers. | Waiting room of the Primary health care facility in Jengre. | Parents and caregivers of children under 5 years’ old | **150 parents/ caregivers from a settlement split into 8 groups.** | **Group training:**  Training consisted of 3 sessions for each group of caregivers with each session lasting 45 minutes to 1 hour.  Part 1 was what is malaria and its causation and transmission.  Part 2 was recognition of uncomplicated malaria and danger sign in children and actions to be taken at home.  Part 3 was about treatment and prevention of malaria | 150 women were tested for baseline data done with a semi structured interview, this data was used to make the intervention.  Post intervention of all 150 parents and caregivers who attended the intervention were given a questionnaire (semi-structure interview) similar to the baseline data  Response to knowledge questionnaires were scored, each correct response received 2 while an incorrect cost received 0. | Knowledge  Medication Dosing Skills | - Statistically significant improvement was seen in the first action taken at home after the onset of fever, first line treatment option and treatment given (p = 0.03 and p = 0.04 respectively) - Time interval between onset of fever and first treatment action did not change significantly (p = 0.51) - There was significant change in second line treatment option (p = 0.02) - Dosage of chloroquine given by the mothers did not change significantly (p = 0.33) - States that tepid sponging and paracetamol the good response while cold baths, plenty of fluids and not action are the incorrect actions. | Measurement tool not in detail  Confounders not addressed  Did not cover bias or limitations + generalization  **MOD-HIGH RISK** |
| Considine J, Brennan D.  (2007)  (S9)  **Australia** | Pre-Post study | To evaluate the effect of an educational intervention on discharge advice given to parents leaving the ED with a febrile child | Metropolitan hospital | Emergency Department nursing staff with the aim of increasing parent and caregiver knowledge | **52 nurses**  **22 family’s pre-test and 18 post-tests** | **Peer-to-peer education:**  Intervention of 2x 30 minute tutorials done by ED nursing staff to see if they gave better discharge information  Training ED nurses followed by interviews with recruited parents for post intervention data | 15 pre/post-test multiple choice (aimed at nurses) on knowledge  Structured phone interview (for caregivers) including quality + quantity of discharge advice | Knowledge (both nurses and parents)  Beliefs  Fever Management Skills | - 31 emergency nurses showed a significant increase in factual knowledge following the tutorials; a mean increase of 3.48 correct responses - The number of parents who reported leaving the ED with no advice regarding management of their child’s fever decreased from 59.1% (n = 13) to 13.3% - There was a 38.4% increase in reports of verbal advice and reports of written instructions increased by 69.7%. - Parents who reported leaving the ED having received both written and verbal discharge instructions increased from 0 to 55.6%. - Non-significant 18.2% increase in advice given by medical staff | Convenience sampling used (for language and education/ communication)  Small sample size  Pre-and post-test groups different  Most results self-reported by parents  **LOW-MOD RISK** |
| Considine J, Brennan D.  (2007)  (S10)  **Australia** | Pre-Post study | To examine emergency nurses:  1 – frequency and autonomy of paediatric fever management decisions  2 – factual knowledge about paediatric fever management  3 – knowledge acquisition following and educational intervention | Metropolitan hospital | Emergency Department nursing staff | **52 nurses**  **(48 completing pre-test and 31 of these completing all MCQs)** | **Peer-to-peer education:**  Intervention of 2x 30 minute tutorials done by ED nursing staff to determine if knowledge of fever and where to acquire information had improved | 2 versions of a validated 15 pre/post multiple choice questionnaire (aimed at nurses) | Knowledge  Knowledge acquisition  Fever Management Skills | - Significant increase in factual knowledge going from a median test score of 7/15 to 10/15 (p < 0.001) - There was no significant difference between level of qualification and increase in test score (p = 0.054) - Knowledge acquisition was unaffected by the tutorials - Nurses who reported independent decisions related to medication administration also had greater emergency nursing experience (p = 0.007), more likely to hold Clinical Nurse Specialist appointment (p = 0.017) and more likely to have a post-graduate qualification (p = 0.006). - Nurses with independent medical decisions were more likely to have higher pre-test scores (p = 0.022) | Convenience sampling used (no power calculation or eligibility criteria)  Attrition not specified in detail  Small post-test sample size  Cofounding factors not addressed  **LOW-MOD RISK** |
| Considine J, Brennan D.  (2006)  (S11)  **Australia** | Pre-Post study | To examine:  1 – the opinions of emergency nurses regarding fever  2 – the effect of an evidence based program on the opinions of emergency nurses regarding paediatric fever | Metropolitan hospital | Emergency Department nursing staff | **52 nurses**  **(48 completing pre-test and 31 of these completing all MCQs)** | **Peer-to-peer education:**  Intervention of 2x 30 minute tutorials done by ED nursing staff focused on their opinions on fever management and best practice | 33 item survey  Included:  -fever 10 items, -antipyretics 13 items  -convulsion 10 items | Knowledge (fever, antipyretics and convulsion)  Beliefs | - Nurses who agreed that temperature in children is often unrelated to severity increased by 22.6% (*p* = 0.020) and there was a 35.5% increase of nurses who agreed that temperatures below 41 ◦C may not be harmful (*p* = 0.001). - nurses who agreed that nurses also share fever phobia increased by 38.7% (*p* = 0.003) - The number of nurses who agreed that nurses determine when Paracetamol administration should occur increased by 35.5% (*p* = 0.003). - Pre-test data showed that 19.3% of nurses agreed that temperature alone is an indication for anti-pyretic administration and 25.8% of nurses agreed that antipyretic medication should be given to all febrile children with a temperature higher than 38.3 ◦C. However, no nurses agreed with these statements following the education program (*p* = 0.014, *p* = 0.003). - There was a 29.1% decrease in the number of nurses who agreed that it is important to aggressively treat fever in children with a history of febrile convulsion (*p* = 0.013). - The number of nurses who agreed that one third of children who have one febrile convulsion will have another within six to twelve months increased by 35.5% (*p* = 0.008). There was also a 25.8% increase in the number of nurses who agreed that positive family history was a risk factor for febrile convulsion (*p* = 0.029) and the number of nurses who agreed that febrile convulsions generally occur in the first 24 hours of illness increased by 19.4% (*p* = 0.001). | Convenience sampling used (no power calculation or eligibility criteria)  Attrition not specified in detail  Small post-test sample size  Cofounding factors not addressed  **LOW-MOD RISK** |
| Cropley L  (2004) (S12)  **United States** | Quasi-experimental pre-post study | To examine the effect of health education interventions on mother’s treatment seeking behaviours for their child’s malaria fevers | Refugee communities in Belize central America | Mothers of young children between 6 months and 5 years old | **402 households (4 communities were in the intervention and 4 in the control with approximately 75 households per community)** | **Group training, one-on-one sessions and material dissemination:**  Three types of education materials were used to communicate the message: a pamphlet, a poster and a malaria post sign  Following development of the materials, workshops were conducted for district health team members, living in intervention villages. At the workshop conclusion, trained personnel were expected to address local beliefs and use local terminology and disease concepts in providing health education at the village level through one-on-one discussions, informal group meetings and material dissemination. | Post intervention survey 6 months after  Monthly reports and observation records were collected and reviewed  Communities were visited weekly to observe health education performance; Voluntary Collaborators (VC) homes and key life path points such as community centres and schools were observed for the presence of materials. | Fever Management Skills  Knowledge  Health service utilization | - Of 400 respondents, 50.5% (202) reported at least one fever in the previous 3 months and of these 37% (148) reported it with at least 1 other symptom - No significant difference was found for either fever or malaria incidence in the intervention and control groups - Of the 148 mothers who reported a case of fever within the last 3 months, 75.6% (*n* = 65) of mothers in intervention group met the criteria for positive treatment-seeking behaviours as opposed to 22.6% (*n* = 14) in control group (p < 0.001). - Of the mothers who reported a case of malaria within the last three months, 84.1% (*n* = 37) of those in intervention group met the criteria for performing positive treatment-seeking behaviours for malaria, as opposed to 37.9% (*n* = 11) for those in control group. (p < 0.001) - Mother in the intervention had much higher exposure to the sign, poster and pamphlet (p < 0.001 for all 3 materials) - Was a significant difference between positive treatment seeking behaviour for fever and exposure to the sign and the poster (p < 0.001 for both) but not the pamphlet - Results were significant of positive health seeking behaviours for fever accompanied by chill (p ,0.001) but not convulsions - Only 24.3% (36) sought treatment from a VC for a child with fever and 57.5% (42) for a child with malaria (knowledge of treatment was high and improved but not enough to make the mother take the child to be treated by a VC) | Quasi-experimental pre-post study  Data measurement tool not described  Confounders not specified  Bias not addressed  Possible selective reporting  **HIGH RISK** |
| Cunningham A, et.al  (2005) (S13)  **United Kingdom** | Pre-post study | To report on the effectiveness of a clinical initiative designed to increase the detection of UTI’s in children under 2 years old | Primary health practices | Primary care practices in the Cardiff area | **6 primary care practices**  **(with comparison data drawn from 47 practices in the same area)** | **Group training sessions:**  It comprised both educational and service development components. Participating practices were given practice-based education in the form of individual or small group seminars  A research nurse kept regular (4-8 weekly) contact with each participating practice to maintain awareness  They were also offered a range of literature, equipment and disposables which they could select as required for their practice.  (Parent information leaflets, urine collection bags, clintek machines and dipsticks, microscopes with counting chambers + instructions, templates for clinical pathways and fast track referrals) | Audits and measures on the number of urine test send to labs for testing as well as referrals and diagnosis with UTI’s were compared | Health service utilization | - At baseline, the intervention practices sent more samples and detected more UTIs than the comparison practices but these differences were not significant. - The intervention produced no statistically significant change in the detection of UTIs, or in the rate of sending samples to the lab - While the initiative encouraged practices to send more samples on young children, and the number of samples increased, this was not statistically significant | Selection bias of locations  Attrition of 1/6 locations very early  Bias and limitations not assessed  1 of the 6 practices dropped out at the start  **HIGH RISK** |
| De Vos-Kerkhot E, et.al  (2014) (S14)  **Italy** | RCT | To assess the impact of a clinical decision model for febrile children at risk of serious bacterial infection attending the ED | Dutch university hospital | Febrile children aged 1 month to 16 years old attending the paediatric ED | **439 febrile children**  **(219 intervention and 220 control)** | **Group training sessions:**  Febrile children were randomly assigned to the clinical decision model or usual care.  The clinical decision model included clinical symptoms, vital signs, and C-reactive protein and provided high-low risks for pneumonia and other SBI’s  Instruction use of the clinical decision model was given, all ED staff were informed by email, and posters and practice cases were given. During the trial period feedback and teaching session were conducted | A central logbook collected data from all participants  Data performed on all lab tests, diagnostic tests and treatment or follow-up were registered in the computer based hospital information system. | Health service utilization | - The discriminative ability of the CDM was 0.83 for pneumonia and 0.81 for other SBI’s - Compliance to the recommendations of the CDM was high with only 3 of the high-risk patients not receiving recommended diagnostics for either pneumonia or SBI’s (done due to over-ruling by the physician). - In 28 low risk patients without clear other focus, no urine dipstick test was done leading to it having a compliance of 86% (188/219) - In 60% (25/42) of all children allocated to the intervention group chest-radiography was done but no pneumonia was diagnosed. This percentage was not significantly different from the 57% (16/28) false positives of the control group (p = 0.84). - In 67% (12/18) of all children in the intervention group urine-culture was collected but no UTI was diagnosed (false positives), but did not differ from the control group (53%, 9/17) (p = 0.41). - The clinical decision model performed similarly between both study groups with regard to false negatives - The median length of stay did not differ between both intervention and control group (1h57min vs 1h54min) - In the intervention group, less full blood-count tests (14% vs. 22%, p <0.05) and more urine-dipsticks were done correctly according to current guidelines (71% vs. 61%, p <0.05). - Overall treatment with antibiotics did not differ substantially between study groups (23% intervention and 27% control p = 0.30) | Allocation concealment not possible  Blinding of outcome assessors not specified  **LOW RISK** |
| Edwards H, et.al  (2007)  (S15)  **Australia** | Quasi-experimental pre-post study | To evaluate the effectiveness of a peer education programme on nurse’s anti-pyretic administration to febrile children | Metropolitan hospitals | Level 1 +2 paediatric nurses | **77 nurses in the experimental and 45 in the control** | **Peer-to-peer education:**  Group was given 4x 1-hour peer to peer education sessions  Followed nurses over an 8-month period | Survey with 84 items across 3 sections containing knowledge attitudes and practice  Chart Audits  Pre-test, post-test then latency test | Knowledge  Attitude  Influences  Medication Dosing Skills | - No significant differences in nurse’s attitudes between experimental and control, or within groups, or across three data collection points. - Experimental group nurses had a reduction and more realistic perceptions of control over antipyretic administration than the control group post PEP (p=0.05) - Reduction in normative influence in the experimental group when compared with the control group post PEP (p < 0.01). - Temperature at which experimental group nurses administered antipyretics in post-test data was significantly higher than in pre-test data (p = 0.01). Positively influenced anti-pyretic administration practises - Experimental group nurses’ intentions to administer an antipyretic to the next febrile child cared for were significantly less than control group nurses when post-test (p = 0.01) and latency data (p < 0.01). | Location choice (hospital) not randomised  Outcome assessors not blinded  Only 75% attendance to intervention  High attrition with 56-78% response rates  **MOD- HIGH RISK** |
| Edwards H, et.al  (2007)  (S16)  **Australia** | Quasi-experimental pre-post study | To evaluate the effectiveness of a peer education programme in developing paediatric nurses evidence based knowledge and attitudes towards fever management and the sustainability of these changes. | Metropolitan hospitals | Level 1 +2 paediatric nurses | **77 nurses in the experimental and 45 in the control** | **Peer-to-peer education:**  Group was given 4x 1-hour peer to peer education sessions  Followed nurses over an 8-month period | Survey with 84 items across 3 sections containing knowledge attitudes and practice  Chart Audits | Knowledge  Attitude  Influences  Medication Dosing Skills | - Experimental group nurses report significantly more overall knowledge in latency data compared to pre-test (p < 0.01) - Experimental group nurses were significantly more knowledgeable about physiology of fever than the control group at post-test (p < 0.01) and latent (p = 0.01) data. Also in experimental groups, there was significantly more knowledge in the latency over the pre-test (p = 0.05) data - Experimental group nurses were significantly more knowledgeable about fever management than control group nurse in latency data (p = 0.01). Also in experimental groups, there was significantly more knowledge in the latency over the pre-test (p < 0.01) data - No significant difference in anti-pyretic dosing between groups and data collection points - Experimental group nurses showed significantly more positive attitudes on the post-test (p < 0.01) and latency (p < 0.01) data than control group nurses | Location choice (hospital) not randomised  Outcome assessors not blinded  Only 74% of nurses attended 1 or more sessions  56-78% response rates in experimental groups with 40-52% in the control  **MOD- HIGH RISK** |
| Eriksen J, et.al  (2010) (S17)  **Tanzania** | RCT (cluster) | To develop an intervention to improve first line case management of malaria in under-five children through primary caretakers in collaboration with local women groups and existing health centres and to evaluate its feasibility and effectiveness on anaemia, fever and malaria prevalence. | Mkuranga district in Tanzania. | 10 wards chosen from 31 wards in the Mkuranga district and from them 1 village from each of those wards was recruited for the study (all homes in each village were used) | **All Health Workers were trained, 36 Women’s leaders were trained**  **Pre-intervention 1928 under 5’s tested and post intervention 2199 under 5’s tested**  The women were trained to treat children with SP as a single dose by age, as specified by the Tanzanian national treatment guidelines. And referral the child to the health centre. | **Group training sessions:**  Followed a training of trainer’s approach:  1: Health worker in all health facilities in those areas were trained consisting of 4 days of lectures combined with presentations and case studies  2:36 women leaders (selected by both community and research team) were then trained by both the previous health workers and the research team (same format as above but more focus on identifying fever cases that should be treated as malaria)  3: Village leaders were informed of the intervention and they met with women leaders to discuss the intervention with the community  4: every 2 weeks’ women leaders visited by HW to order new drugs  5: health workers assess women leaders with a standardized checklist and collect forms about diagnosed and treated cases  Training of staff was in 2004 April-May where the intervention went from may2004 to February 2005 | Pre-intervention survey was given to all households in the district (with children under the age of 5 years old) (mainly focused on demographics, economics and care seeking behaviours)  Only 1 child per household was included and this child was taken to a ‘field lab’ where a clinical officer and a nurse reviewed the child’s health condition and interview them again (different survey).  Here they were tested with:  1: Thick blood smear  2: Filter paper for detection of Haemoglobin levels | Health service utilization  Fever Management Skills | - Each woman leader saw on average 105 (23-217) children during the 12-month period. Over 94% of these were children with the primary concern of fever - A significantly lower prevalence of anaemia (p<0.001) was observed in the post intervention survey compared to the pre-intervention period (0.5% vs. 37%) and the decrease was significantly larger in the intervention (from 43.9% to 0.8%) compared to the control (30.8% to 0.17%) group (p=0.038). - Overall, fever prevalence (axillary temperature > 37.5ºC) and reported fever episodes during the last 48 hours were also significantly lower and mean body weight was significantly higher during the post intervention compared to the pre-intervention survey (p<0.001), but there were no significant differences between control and intervention groups (p=0.204). - Pre-intervention, 25.8% of the study children had Plasmodium falciparum parasites detected in their blood smears. Unfortunately, due to logistic difficulties, we could not perform a validation of the parasitaemia results post-intervention. We therefore chose not to present the post-intervention results. - Multivariable analyses were performed on anaemia, adjusting for age of mother, education of mother, and weight for-age z-scores of the child. Pre-intervention, only a low weight-for-age z-score of the child was independently associated with anaemia (p<0.002). There were too few cases of anaemia post-intervention to do the same analysis - No significant effects of the intervention on increased body weights (p = 0.436) - Shown to be feasible under programme conditions for lowering anaemia (this was also not fully relevant to the initial talks of fever and malaria which were not very well covered in results). However little of this could be directly contributed to the intervention | Same cohort not followed pre/post  Blinding of outcome assessors (or participants) not specified  Missing & not reported blood specimens in laboratory tests  **MOD RISK** |
| Fieldston E, et.al  (2013) (S18)  **United States** | Quasi-experimental pre-post study | To test the impact of and educational and training intervention about management of common childhood illness on caregiver knowledge and health service use by an index child | Primary care offices | Parents/ Caregivers of children aged 7 months to 5 years recruited from 4 urban primary care centres and Children’s Hospital of Philadelphia | **32 parents** | **Group training sessions (didactic):**  The intervention was a 90-minute educational activity consisting of didactic teaching, skills demonstrations following a participatory curriculum developed with adult learning principals in mind.  This was done by paediatric nurses from the ED  Addressed fever, colds and mild trauma | Parents given a questionnaire before, after and 6 months after the intervention.  It was a 19 item MCQ survey with 9 items on fever, 4 on cold and 6 on trauma.  Medical records of the child 6 months before and after the intervention were also collected (focusing on ED visits, calls to the clinics, and physician visits) | Knowledge  Medication Dosing Skills  Health service utilization | - Knowledge score significantly increase from pre-to post intervention (55% to 77% p < 0.001). This occurred in all 3 areas of fever, colds and trauma - On follow-up 6 months later, only 20/32 parents could be contacted, of these scores the mean declined from 79% to 71% (p = 0.031), however this was still and increase from pre-test scores 61% (p = 0.015). - Health service use over the 6-month study period did not significantly change (while they value moved both up and down) However after hour calls rose significantly from 0.33 per patient to 1.46 per patient (p = 0.047) - Previous level of health service utilization was a positive predictor of post intervention health service utilization (p < 0.01) - Employment status was associated with post intervention use of evening and after hour phone calls to the primary care centre (p = 0.002) | Small sample size  Testing was not controlled for seasonal illness changes  Demographics of black, unemployed 1/5^th^ less than 11 grade education and 1/5^th^ higher than 12 grade education  **LOW-MOD RISK** |
| Hart L, et.al  (2016) (S19)  **Canada** | RCT | Aimed to compare caregiver’s knowledge acquisition and satisfaction with 2 novel Web-based tools (WBM and ROW) for childhood fever education | Paediatric Emergency department at a children’s hospital in Ontario Canada | Caregivers of children aged 0-17 years presenting to the ED with the chief complaint of fever or had a measured temperature by triage nurse greater than 38°C | **77, 79, and 77 caregivers included in the WBM, ROW and SOC groups respectively** | **Computerized training tutorials and guidelines:**  Participants split into 3 groups:   - Web Based tools (WBM) - Read only website (ROW) - Standard of Care (SOC) (the control)   WBM group used a touchpad to follow an interactive set of components including presenting buttons, information, computer feedback on responses and clicking to learn more (to name a few)  ROW was a web based document without any intractable elements. It contained identical information to the WBM  SOC consisted of the same information but in paper based documents detailing appropriate care.  All information was the same and included definition, measurement, fever management and when to seek medical attention. The groups got about 20 minutes with the tools | Knowledge was measured at baseline and post-test with an 18-item questionnaire  Was scored between 0-33 with each question worth 1 point (some had multiple parts)  This questionnaire was modified from Chiappini Et.al and was pre-tested for content validity and face validity  Caregiver satisfaction was measured using a survey for Web-based tools with a score from 0-32 points | Knowledge acquisition  Satisfaction | - WBM and ROW groups were associated with significantly greater questionnaire scores compared to SOC alone (p < 0.001) (pre-to immediate post-test). This relationship held even after adjusting for caregiver education levels, number of children, prior antipyretic dosing and health care occupation. - WBM and ROW were significantly greater score in pre-test to delayed post-test, however loss to follow-up attrition leaves this invalid - Patient scores on satisfaction were rates as followed (score out of 32):   22.6 (WBM) > 20.7 (ROW) > 17.0 (SOC) | Was no standardised script, and patients was asked to refrain from seeking further information not assigned to their group  High loss of follow-up post-test 50% attrition  Low satisfaction discussion  **LOW RISK** |
| Hu F, et.al  (2016) (S20)  **China** | Pre-post study | Aimed to establish the evidence based management of fever in the ED of Children’s Hospital of Fudan University by implementing the best available evidence for clinical practice | Emergency department of the Children’s Hospital of Fudan University, China. | All nurses which worked at the hospital were audited and part of the study  Also, children presenting with fever and their caregivers were utilised for the audit during the time period | **Included:**  **35 Nursing Staff**  **50 children and their carers** | **Group training, one-on-one sessions and material dissemination:**  After findings were collected from the pre-audit, strategies were put in place such as:  A training workshop given to the nurses regarding fever management, recognition, assessment, treatment and medication.  Also, the traffic light system was adapted from NICE guideline and integrated into their current records  For caregivers, educational +cartoon posters and pamphlets where developed for dissemination along with a video being developed for education.  For caregivers:   - Video   Material dissemination | Data was measured and collected using a pre/post audit measured with:   - Checking the nursing procedures - checking the nurses training signature sheets - Checking the nursing record of feverish children - Checking patient case records - A caregiver survey | Knowledge  Fever management skills | - Before training baseline average scores of knowledge was 54.5 for caregivers and 67.6 for nurses. This increased to 83.7% for caregivers and 90.9% for nurses. - Using correct methods to measure temperature improved from 86% compliance to 98% compliance - The number of staff and parents having received some form of education of fever knowledge and management had increased from 0% to 100% post audit - Increase in nurses use of the assessment tool from 0% to 100% post audit - Number of children observed in hospital seen as high risk by NICE standards increased from 0% to 98% - Nurses and parent completed the baseline questionnaire and responded to the same questions after the training and education - Unsure how they measured caregiver education (implying they gave new caregivers that arrived to the hospital education) | Audit may not have measured the same patient/caregivers therefore data may be inconsistent  Include/exclude criteria not shown  Unsure of how recruitment was done  Seasonal changes not accounted for  Details of the training method and type unclear  **HIGH RISK** |
| Huang M, et.al  (1998) (S21)  **Taiwan** | RCT | To characterize the effects of an educational program on knowledge, attitudes, concern and first-aid measures among parents with febrile children | 8 major referral hospitals and 3 outpatient clinics in Taiwan | Parents/ Caregivers who took their children to one of the 8 major referral hospitals and 3 outpatient clinics in Taiwan | **129 families (65 in intervention and 64 in the control groups)** | **Group training sessions:**  Intervention was a 2-hr educational program consisting of 3 sessions  It was held in a regular monthly basis over the time period  Both groups completed identical post-test questionnaires. Control group was given the intervention after post-test data. | A six-part structured questionnaire was developed:  **1**, demographics  **2**, past experience  **3**, parental knowledge (11 T/F Q’s)  **4**, attitude towards FC (10 5 point Likert scale Q’s)  **5**, Concerns about FC (10 5 point Likert scale Q’s)  **6**, anticipated measure for next FC episode (15 Y/N Q’s)  Parents completed a pre-test by mail 3 weeks before attending the intervention | Knowledge  Fever Management Skills  Attitude  Health service utilization | - Parents of children with more than 1 Febrile Convulsion (FC) had better pre-test results (47% vs 37%) regardless of control or intervention group (p = 0.03) - Significant improvement was seen in all 3 categories of knowledge in the intervention group compared to the control (p < 0.0001) - Significant improvement was found in the 3 categories of attitude on FC in the intervention group compared to the control (p < 0.0001). However, there was only slight change concerning attitude concerning body temperature measurement. - Significant improvement in the category of further FC episode in the intervention group over the control (p < 0.0001) - Most recommended and non-recommended behaviours towards FC episodes and service utilisation were significantly improved after the intervention. | Randomisation of locations and participants not stated  Blinding of participants and assessors not described  **HIGH RISK** |
| Huang M, et.al  (2002) (S22)  **Taiwan** | Quasi-experimental Pre/post-test design | To evaluate the effects of educational interventions on parental practices for recurrent febrile convulsions (FC) | Major cities in Taiwan | Parents of children aged between 6 months and 6 years with a febrile convulsion or recurrent episodes who visited the emergency room of 1 of 8 major referral hospitals or 3 outpatient clinics | **326 parents/ caregivers (130 intervention and 196 control)** | **Group training sessions:**  Two alternative interventions, a mailed pamphlet or a 2-hour parental education program  Was split into 3 sections:  1: a paediatric neurologist explained the nature of FC and its outcome (25min)  2: paediatric nurse explained recommended (and non) practices for children at home (included demonstrations and rehearsals were conducted (25 min)  3: nursing member lead a mediated parent discussion (50 min)  Program undergone monthly | Measured with a behaviour questionnaire to gather parental practices made up of 15 items in a true/false format  Data was collected pre-intervention, then post intervention at 3, 6, 12, 18 and 24 months. | Knowledge  Fever Management Skills | - Within the 2 years, 78 of the 326 FC children had recurrent FC’s - No differences in baseline practices used during the initial seizure between the intervention and the control group as well as the sample 78 which had recurrent FC’s - The intervention group demonstrated significant improvements for recommended practices in dealing with recurrent FC. Similarly, non-recommended practices also decreased significantly - Fewer parents reported to be too overwhelmed to do anything (36.1% to 8.3%) (p < 0.001) - Compared to the control, the intervention group demonstrated significant improvement in parental practices. Similar for non-recommended practices such as less rushing the child to hospital (OR=6.1, and refrain from putting devices in the child mouth (OR=3.9) - Compared to control, the intervention group demonstrated significant improvement in observing manifestations and duration of a child seizure (OR=8.4) and positioning of the child (OR=3.7) | Non-equivalent groups sued  Attrition of 16-17% on follow-up  Allocation not described  Blinding not used  **MOD RISK** |
| Ismail S, et.al  (2016) (S23)  **USA** | RCT | To determine if innovative video instructions for fever and Closed head injury improved caregiver’s comprehension of the child’s diagnosis and follow-up care | Paediatric emergency department during discharge | Parents/ caregivers in the Emergency Room for child with a primary complaint of Fever of Head Injury | **31 parents (15 control, 16 intervention)** | **Video:**  Control had standard discharge information given verbally and written.  Experimental group was given a short video in containing instructions and guidelines in regards to fever management or a closed head injury | 9 question and 18-point questionnaire  Combination of True/False and multiple choice  Addressing diagnosis and disease process  Small informal interview on satisfaction | Knowledge  Fever Management Skills  Satisfaction | - Fever group had statistically significant difference in post-test scores for the intervention group (median score 88.89) over the control group (median 72.22) –Age, sex and education did not matter - No difference between the intervention and the control group with follow-ups to their child’s primary care physician/ paediatrician - 92% agreed the videos provided additional information and liked the video information in addition to the standard discharge - Intervention group with less than HS education (Mdn = 89.47) and more than HS education (Mdn = 88.89) had similar test scores (p = 0.13), whereas those in the control group with less than HS education (Mdn = 66.67) had significantly lower test scores than those with more than HS education (Mdn = 77.78; | Randomization sequence not specified  Blinding not described in detail  Small sample size  Predominantly female and African(black) for the demographic  **MOD RISK** |
| Jeong YS, Kim JS  (2014) (S24)  **South Korea** | Quasi-experimental Pre/post-test design | To compare the effects of a blended learning program of fever management for paediatric nurses with a face-to-face one using the Theory of Planned behaviour | Korean metropolitan city | Paediatric nurses  Recruited from children’s hospitals | **59 paediatric nurses (30 in intervention and 29 in the control groups)** | **Blended learning (2 sessions Face-To-Face + 3 online sessions) vs 5 sessions of face-to-face (FTF) learning:**  Intervention was a blended-learning program split into 5 modules. It comprised of 2 face to face meetings (the first and last session) lasting 90 minutes and 3 online learning sessions lasting 20 minutes each  Control was 5 face to face sessions (1 per week)  In both groups, face-to-face sessions had lectures, discussion and Q&A | A pre-test and post-test survey was self-administered  Both surveys were identical and contained:  - 20 MCQ on fever knowledge  - 33 items (Likert scale) on fever management attitudes  - 3 items on normative influence  - 4 items on perceived control of fever management  - 2 items on intent to uses anti-pyretic  2 items were satisfaction with the education program | Knowledge  Attitudes  Influences  Medication Dosage Skills | - No significant baseline differences for all variables - For both groups, post-test results indicated knowledge of fever, fever management, attitudes and intentions for anti-pyretic use showed statistically significant positive changes (p = 0.001) - For fever knowledge in the blended group mean scores increased (10.90 to 13.00) similar in the face-to-face (9.80 to 11.52) (P < 0.001) - For attitude toward fever management in the blended group mean scores increased (99.33 to 108.80) similar in the face-to-face (99.00 to 107.55) (P < 0.001) - For intention for anti-pyretic use in the blended group mean scores decreased (11.97 to 9.67) similar in the face-to-face (12.14 to 10.57) (P = 0.001) - Normative influence and perception of control changes slightly however was not seen as significant. (p = 0.36 and p = 0.67 respectively) - Satisfaction with both groups was high, however blended learning (4.86, 4.83) was significantly higher than face-to-face (4.48, 4.48) (p = 0.004) | Non-equivalent groups sued  Blinding of assessors not described  Small sample size and intervention time  **MOD RISK** |
| Kelly L, et.al  (1996) (S25)  **United States** | Descriptive Comparative study | To examine:  1: the level of knowledge that the caretaker demonstrates regarding the implications that fever has on their child’s welfare?  2: what clues or child behaviours are initiated by caretakers  3: the level of knowledge caretakers demonstrate regarding appropriate fever management  4: the effectiveness of an educational intervention in relation to knowledge and fever management | Done at 5 suburban primary health care centres and practises | Caretakers of children between the ages of 2 and 5 years’ old | **86 caretakers (50 of these participated in the follow-up interview)** | **One-on-One sessions:**  The distribution of the instruction sheet and the correction of Information constituted the educational intervention for this study.  At the completion of the 15- to 20-minute interview, subjects were given the printed instruction sheet regarding fever management, and it was reviewed with them. If caretakers made statements regarding fevers or the management of fever that indicated a knowledge deficit, the investigators provided accurate information. | 13 item (approx. 20 minute) questionnaire consisting of open ended questions  3 were on caretaker assessment activities  4 were on methods and rational of fever management  5 were on selection of treatment modality and medication dosing  This was followed up by phone interviews 2-4 weeks later repeating the 20-minute questionnaire | Knowledge  Medication Dosage skills | - The fever level at which anti-pyretics were dosed was shown to have a significant association after the intervention (p < 0.022) - There were no significant differences were present in the level of fever at which antipyretics were administered before and after the educational intervention (p = 0.91). - Therefore, for this study the intervention was not effective in terms of the level at which to medicate a fever. - A significant difference was obtained that showed that the educational intervention did improve anti-pyretic dosing accuracy (p = 0.04) between pre-and post-intervention assessments - Of those who measured a low dose pre, 55% improved. - Of those who measured a high dose pre, 100% improved - Of those who measured a correct dose pre, 16/17 provided a correct dose | Attrition of 40% for follow-up interview  Comparison details very light  Small sample size  Little bias discussion  Talked about medication dosing by body weight  Lot of the information is getting the opinions and behaviours and knowledge levels but not comparing them, low depth  **MOD RISK** |
| Light P, et.al  (2005) (S26)  **United States** | Descriptive correlational study | To identify whether parental advice for home care by telephone triage paediatric nurse led to a change in location of care from the ED or medical doctor’s office to home care for children aged 12 to 24 months with an uncomplicated febrile illness | Data from a Children’s Care hotline database | Parents of children aged 12-24 months calling the nurse hotline with uncomplicated febrile illness as the primary problem | **110 parents/ caregivers** | **Hotline phone call records:**  Calls to the hospital are forwarded through the Children’s Careline. Standard protocol is followed by the nurses accessed through the hospital computer system and given to callers.  On analysis of patients background, history and symptoms, the nurse chose the correct protocol and directed the caller for appropriate treatment  Data was collected from the database over a period of 2 years | Measured with computerized charts which provided the history of the call and the protocol which was followed | Health service utilization | - Of the 110 parents given home care advice from the hotline, 22 of the parents brought the child to the physician and 88 followed the advice given - 53 of 74 parents whom initially thought they needed a visit changed to home care - Most parents who wanted a doctor’s office visit kept their child at home at the advice of nurses (48/67) - Chi tests demonstrated a high-low to low-moderate correlation between the original preference of a parent regarding location of care (Emergency Department or doctor visit) for their child and the change to home care after receiving the triage advice from a nurse (p < 0.05) | Limitations and bias control not included  Variables explained in discussion that were not included in study  Done as a retrospective database  **MOD-HIGH RISK** |
| Marsh V, et.al  (1999) (S27)  **Kenya** | Observational pre/post Study | To examine the feasibility and measure the likely impact of training shop keepers in rural Africa on community drug use | Shopkeepers stores in the Kilifi district Kenya. | Shop keepers on the rural coast of Kenya | **46 shop keepers from 23 shops (serving an approximate population of 3500 customers)** | **Group training, one-on-one sessions and material dissemination:**  All shopkeepers were trained in a series of 3 workshops each lasting 3 days  Emphasis was placed on active participation, practical training and skills evaluations  Focus put on brand name drugs and using 2 types of materials to give information to purchasers on how to use the drugs, dosage charts for chloroquine/aspirin/paracetamol based drugs and sets of stamps on how to dose chloroquine in children of different ages  After the workshop, 1-2-hour individual training sessions were held in the shop to allow direct observation.  A 2-day refresher workshop was help after 6 months  All tests done from Dec 1995 to January 1997  Measurements taken in 2 rounds (June/July 1996 and Dec/Jan 1997) | **BASELINE**:  Baseline data collected by monitoring the 14 busiest shops in the areas (for 9 days) observing the sales of anti-malarial and anti-pyretics with the purchaser being interviewed outside the store.  Shopkeepers also informally interviewed on their current knowledge, attitudes and practices related to malaria  Caretakers of children 6 months to 10 years old were interviewed discussing symptoms of the illness, health seeking behaviours and drug use.  **POST:**  Changes in percentage of sales where anti-malarial or anti-pyretics were purchased, total numbers of chloroquine tablets purchased and reported use of shop bought drugs. | Sales of medications  Health service utilization  Medication Dosing skills  Perceived Confidence | - Post intervention in both time points measuring the amount of purchased antimalarial drugs significantly rose (n=99: 34.3% to n=199: 84% to n=119: 79.3% at pre, post1 and post 2 respectively) (p < 0.000001) - Post intervention in both time points measuring the number of patients adequately dosing antimalarial drugs significantly rose (n=31: 31.8% to n=196: 82.7% to n=107: 89.9% at pre, post1 and post 2 respectively) (p < 0.000001) - Post intervention in both time points measuring the amount of advice given to patients on antimalarial drugs significantly rose (n=2: 2% to n=186: 93.5% to n=116: 97.5% at pre, post1 and post 2 respectively) (p < 0.000001) - Both the purchasing of anti-pyretics only or the purchasing being a child significantly dropped in both time points after the intervention (p < 0.000001 for both cases) - Adequate chloroquine use increase significantly over the 2 post intervention time points with a significant reduction in overdose and under doses (p < 0.000001 in all cases) - Number of single doses of chloroquine also significantly lowered with a significant rise in the correct dose given over 3 days (p < 0.000001 for both cases) - Use of aspirin significantly lowered across all time points (236 vs 128 vs 70). Of those still choosing to use aspirin, safe doses were significantly higher from baseline up to post intervention (78% vs 93.8% vs 97.1%) with a p < 0.00001 and p < 0.0002 in the post readings at 6 and 12 months respectively - Number of overdoses in aspirin also significantly reduced as a result (22% vs 6.3% vs 2.9%) (p < 0.0001 and p< 0.0002 at 6 and 12 months respectively) - Community members interviewed stated they strongly supported the aims of the training program and reported an increased confidence in the information offered by shopkeepers - Shopkeeper perceived benefits were increased community status, increased knowledge in treating family members, increased confidence and increased profitability. - Stated in results and discussion that not all data was published | Demographic studies, training program and results published elsewhere  Study design not stated  Small sample size  No selection criteria (other than the biggest 23 shops)  Little detail on bias minimisation and limitations.  States that not all data was published  **HIGH RISK** |
| O’Neil-Murphy K  (2001) (S28)  **United States** | Quasi-experimental pre/post-test study | To determine:  1: If a fever education program (interactive or written) reduced parents fever anxiety.  2: If an interactive fever program was more effective as a teaching style than standard written material alone  3: If a fever program increases parent fever home management and reduce return ED visits | Urban paediatric emergency department | Parents/ Caregivers who presented to the Emergency Department with primary complaint of ‘fever’  Child was 3 months – 5 years with no serious coexisting illness | **87 parents (44 in intervention and 43 in the control groups)** | **One-on-One sessions:**  One of 2 programs given:  Interactive fever program which provided a discussion, review of a fever pamphlet, discussion of parent’s questions and concerns and instruction or correct thermometer use.  Stand fever program provided a pamphlet that contained fever definitions, information on home management and correct temperature taking.  Odd days got the standard treatment while even days got the interactive treatment.  Done over 9 months | Pre-test parents asked to rate anxiety on a 5 point Likert scale  After their intervention, they again asked to rate their anxiety.  Telephone follow-up at 2 and 8 weeks’ semi-structure regarding repeated fever episodes, return to the ED, home management practices and anxiety. | Participant Anxiety | - No significant difference in how parents identified fever in a child between the 2 groups. - Excess of 40% of parents in each group reported moderate to high levels of anxiety related to their child’s fever at the ED. After either fever education, parents in bother intervention and control group reported reduced self-reported anxiety by 85% and 83% respectively. - By 2-week follow-up, 86% of the control group and 50% of the intervention felt less anxious when their child has a fever. - Previous studies showed 48-78% of subjects were unable to correctly read a thermometer. However only 16% of the control and 7% of the intervention were unable to read thermometer. (results state may be due to large number and colour coded on the thermometer) | Even/odd day randomization  High attrition rates of 15% control and 48% experimental  Only 86% of the control and 52% of the intervention were contracted for follow-up  Blinding unspecified  Doesn’t uses a lot of the data they found (not statistics  Not bias control or limitations  **MOD-HIGH RISK** |
| Pusic V, et.al  (2012) (S29)  **United States** | RCT | To evaluate the educational efficacy of a clinical education model in which the student moves through a sequence that includes immediately reinforcing their learning using a specifically designed computer tutorial | Paediatric emergency department | Medical students | **146 students, 92 were given a tutorial (41 of these were in the intervention ‘in sequence’ group)** | **Computerized training tutorials and guidelines:**  The intervention was 1 of 2 tutorials aimed at factual knowledge on the topic (either oral rehydration or fever WS)  The order of the intervention was as follows:  -Student had patient interaction  - Student then saw preceptor to discuss  -Student underwent tutorial Intervention  -Student wrote up their report | Pre-and post-tests were the same examination for Oral rehydration (6 items) and Fever (10 items)  Questions were a mix of MCQ and constructed responses testing knowledge | Knowledge | - Students who were able to complete a tutorial scored higher on post-test (54.0% vs 37.0%) than those who did not do an intervention - Across all study groups, 146 participants did both pre-and post-tests had an average combined raw score increase from 31% to 48% (was significant at p < 0.001) - There was no difference between the groups doing the tutorial in-sequence and out-sequence for both ORS (61% in vs 63% out) and FWS (56% in vs 55% out) - ANCOVA model showed that in sequence condition had a significant effect on post test score over and above independently seeing the patient or doing the tutorial in isolation. Was statistical significant for in-sequence scoring 19% higher than the comparison group | Allocation concealed up to intervention  Outcome assessors blinding not specified  **LOW RISK** |
| Robinson J, et.al  (1989) (S30)  **United States** | RCT | To evaluate the effectiveness of an audio-visual presentation of fever at increasing parent knowledge about fever, confidence in managing fever at home, and satisfaction with health education services. | Paediatric after hours’ clinic | Parents/ caregivers of children under 13 years of age with the complaint ‘high temperature’ or ‘fever’. | **497 families (247 in intervention and 250 in the control groups)** | **Video:**  Intervention was a 10-minute slide tape of fever given at the clinic before they saw the physician  Both intervention and control were after the visit given a pamphlet that contained and reviewed all major points of the video  Each group was given the survey only once at different time points  In the end, 72 in the intervention group and 73 in the control group did not get a survey at all. | Survey was 10 items and regarding fever knowledge, attitudes and practices. Was a combination of T/F and MCQ.  30 intervention and 60 control filled out the questionnaire pre-test  30 intervention group subjects filled out the survey post-test  Telephone follow-up was done at:  2 weeks (40 intervention and 40 control)  3 months (30 intervention and 30 control)  6 months (47 intervention and 45 control)  Telephone follow-up was also semi-structured and satisfaction was also tested | Knowledge  Satisfaction  Health service utilization  Attitudes | - Intervention group saw immediate improvement if knowledge score after the video intervention averaging only one wrong response compared to the pre-test average of 6 wrong responses (p < 0.001) - At both 2 weeks and 3 months the intervention group showed significantly better post-test score than the control group (p < 0.001 for both). This faded by 6 months with a score of 4.07 for intervention and 5.11 for the control groups (p = 0.07) - Overall average however was significantly better than baseline for the experimental group across all time points (p < 0.05) - Control post-test score also improved significantly (p < 0.005) most probably due to the pamphlet. This improvement was not as large as the intervention group. - In the 30 days after the intervention, there were fewer visits to the clinic in the intervention group than the control group (0 to 8 visits respectively) (p < 0.005). This effect slowly decreases to a final difference of 35% by 7 months (p < 0.05) - By 8 months, intervention families had mad 25.3% fewer visits to the clinic for acute complaints than the control (p < 0.001) - At 2-week follow-up, 100% of intervention group remembered receiving health education while only 80% remembered from the control group. - Intervention group stated they learnt more that they had not known for the education material than the control (73% vs 40%, p < 0.02) - 2 weeks after the intervention, intervention group felt more confident in managing fever at home than the control group (88% vs 55%, p < 0.002) - Almost all (39/40) of the intervention subjects wanted to see more audio-visual health education programs | Random sequence generation not described  Blinding of assessors not described  More parents in the intervention group actually read the extra pamphlet (85% vs 68%)  Bias and limitation not covered in detail  **LOW RISK** |
| Ruvinsky S, et.al  (2013) (S31)  **Argentina** | Prospective longitudinal pre/post study | To assess the effectiveness of a program aimed at improving the use of hospital antimicrobials in patients hospitalized at Hospital Garrahan. | Urban hospital in Argentina | Children 1 month to 16 years old who were taking antibiotic in the hospital were utilized. | **376 patients in pre-intervention and 357 patients in post intervention** | **Peer-to-peer education:**  Intervention was aimed at medical assistants, fellow residents, pharmacists and infectious disease specialists.  The intervention was 20 peer-to-peer discussion workshops were developed based on collected initial data.  Was done to discuss clinical conflicting situations, antibiotic uses and determine recommendations for the hospitalized patient.  From this, management algorithms were developed and disseminated.  Undergone from November 2010 to June 2011 | Clinical data was obtained from all patient’s medical records both pre-and post-intervention. (Chart audits)  This was analysed by 2 specialized paediatricians following guideline to determine if treatment with antibiotics was adequate or not for each situation. | Antibiotics prescriptions  Medication Dosing Skills | - Significant decrease was seen in the inadequate use of antibiotics from pre (35.6%) to post intervention (21.6%) (p < 0.01) - Significant decrease was seen in the inadequate use of ALRTI’s from pre (52) to post intervention (17) (p < 0.01). - Similar results were seen for hospitalized patients who developed a fever without a clinical source (from 25 down to 7 cases of inadequate treatment) (p < 0.01) | No control group  Randomization method not specified  Sources of bias and limitations not covered  **MOD RISK** |
| Sanghavi D, et.al  (2005) (S32)  **United States** | Pre-post study | To design a waiting room educational kiosk that uses interactive, self-guided, computerized tutorials to give anticipatory guidance to parents at the 6-week and 4-month well-child visits, and assess impact on parent knowledge. The intervention required no additional provider time, and automatically printed a summary for  the medical record. | Government funded hospital waiting room | Parents of children receiving healthy well-child visits at 6 weeks and 4 months | **101 parents/ caregivers (52 in control and 49 in intervention group)** | **Computerized training tutorials and guidelines:**  Parents completed a computerized instructional tutorial before being seen by the HCP. The tutorial was 20 minutes and was based on content from ‘Bright futures: Promotion of health habits’ guidelines for 2 months and 4 month visits  Control group only received written materials to review before seeing the HCP | Was a 21-item questionnaire testing mainly knowledge.  Used both MCQ and fill-in-the-blank questions  Covered topics regarding car seat use, fever management, dental care, infant nutrition and basic demographics  Done after receiving the intervention (or control) | Knowledge | - In all tested areas, there was a significant difference in the knowledge of the intervention group over the control group with every section in the intervention group having a higher average score than the control - Total correct responses to the questions was 61% in the control group and 81% in the intervention group (p < 0.001) - Percentage of parents with a perfect score or only 1 question wrong was 2% in the control group and 35% in the intervention group p < 0.001) - **For reasons stated, the fever questions were taken out of the 4-month questionnaire** | Study design not stated  Used different tools to measure post intervention values  Blinding not specified  **HIGH RISK** |
| Sarrell M, Kahan E (2003) (S33)  **Israel** | Pre-post study | To evaluate the impact of a single-session educational program for parents on the approach to febrile illness | Community paediatrician practices | Parents/ caregivers | **156 parents (became the study group for the reinforced session)** | **One-on-One sessions:**  A detailed reinforced one-on-one session with a paediatrician in the practice.  Done after a paediatrician visit for fever treatment | 3-part validated questionnaire split into demographics, satisfaction and knowledge + approach.  The 3^rd^ having 7 items rated 0 or 1 | Knowledge  Fever Management Skills  Health Service utilization  Satisfaction | - 75% of parents correctly defined the lower limit of fever (as >38.5) compared to 46% before the session - 68% correctly initiated treatment for a temperature above 38.5 compared to 30% before the session - 39% still though hand contact was sufficient to measure temperature compared to 54% before - Thermometer use was not significant - 96% responded appropriately to non-pharmalogical treatment compared to 50% before - 43% of the parents correctly assessed when to visit a paediatrician compared to 23% before same trend for ER visits (63% from 38%) - 96% rated mod-high satisfaction for the reinforced session while 78% for the standard lecture - Still promotes tepid bathing and sponging the child, but year of study should be noted | Study design not stated  Not talk of bias or limitations  1 cohort so nor blinding, randomization or control  **MOD RISK** |
| Schriger D, et.al  (2000) (S34)  **United States** | Quasi-experimental interrupted time series | To determine if clinical guidelines imbedded in an electronic medical record improved quality of care while lowering costs of the health care workers of young children with febrile illness in the emergency department. | University hospital emergency department | Febrile children under 3 years and the physicians who treated them | **830 febrile children and their treating physician,** | **Computerized training tutorials and guidelines:**  A computer intervention which searches clinical guidelines through a fever module and provides evidence-informed advice regarding the content of the patient history, physical examination, use of lab testing, interpretation of lab results, administration of medicines, diagnosis and content for after care instruction when patients are sent home  PHASE 1 normal charting was used  PHASE 2 EDECS program was used for febrile children  PHASE 3 hand written charts used exclusively  All physicians given a 15-minute orientation on the software use  Study done from 1992 - 1995 | Quality was measured by looking at 21 essential items needed on each chart and the percentage completed  A computer algorithm calculated No. of appropriate decisions over No. decisions.  Charge data measured even if price fluctuated (used initial prices) | Quality of documentation  Appropriateness of treatment  Per-patient charge for visit | - EDECS was used in 64% of the patients cared for in PHASE 2 (the rest were hand written) - Documentation was higher in phase 2 then phase 1 for 20/21 items. Documentation in phase 2 was higher than in phase 3 for 21/21 items - Overall documentation of the 21 items was 80%, 92% and 74% for Phases 1,2 and 3 respectively. (in phase 2 written charts had 78% documentation while EDECS had 100% leading to the average of 92% which was classified as a significant improvement) - EDECS may have simulated doctors to create more complete charts however it did not change behaviour. Example of and ear blowing examination had a compliance rate of 15%, 22% and 5% across Phases 1,2 and 3 showing that compliance was not always noted. - Overall documentation of aftercare instructions was 48%, 81% and 50% for Phases 1,2 and 3 respectively. The percentage for just written instructions stayed at a constant 48%, 48% and 50%. - Mean total charges were similar across all 3 Phases (216$, 216$, 222$). This was also adjusted for appropriate test and treatments - There were no important differences in utilization or appropriateness of diagnostic tests among EDECS and written charts in Phase 2 | No randomization or blinding of participants or personnel  Chart inclusion criteria now well explained  Confounding bias over time and documentation type  **LOW-MOD RISK** |
| Statile A, et.al  (2016) (S35)  **United States** | Pre-post study | To describe the creation of an innovative paediatric HM curriculum designed to increase learner medical knowledge and their confidence in communicating with parents and families about these topics.  Secondary objective to evaluate the level of innovation of the conference sessions perceived by learners. | Cincinnati Children’s hospital medical centre | Medical students, interns and residents at a Children’s Hospital | **181 medical students and residents in the intervention,**  **17 in fever group**  **32 in wheezing, health care system and bronchiolitis group**  **30 in UTI group**  **38 in child abuse group** | **Group training sessions:**  Intervention was the creation of a HM curriculum consisting of 6 modules.  Each was portrayed in 1 hour conferences given on weekdays in 6 bi-monthly sessions given  All the sessions used a lot of strategies including team-based problem solving, expert presentations and discussions, case debates and pop-quizzes.  Study done from 2011-2013 | Pre-and Post-session survey was 3 parts (3 questions in total).  A write in medical knowledge question, a 5 point Likert scale question regarding perceived confidence of the learners, a 5 point Likert scale question on the level of innovation of the conferences. | Knowledge  Perceived Confidence  Perceived innovation of intervention | - Significantly increase in learner medical knowledge was seen pre-and post-sessions for all individual topics (p < 0.001) - Medical knowledge of Fever/meningitis topic increased from 0% correct to 82% correct responses (p < 0.001) - Confidence in communicating about the topic with families also significantly increased for all topics (p < 0.001) - For fever/meningitis this increased from an average confidence score of 3 to 4 post (p < 0.001) - All sessions were rated highly innovative with median scores of 4 or 5 from every session. | No inclusion or selection criteria  Some participation bias (completed pre-not post)  Not very generalizable  **LOW-MOD RISK** |
| Steelman J, et.al  (1999) (S36)  **United States** | Pre-post study | To determine if intervention would improve parental understanding and management of childhood fever | Keesler USAF medical centre paediatric clinic | Parents /caregivers of children | **93 subject (50 in the intervention group and 43 in the control)** | **One-on-One sessions:**  Study undertaken during infant visits to the medical centre pediatric clinic.  Both groups received a standardized age appropriate slide presentation discussing well-infant care topics in childhood fever.  Intervention group received an additional 10-minute slide presentation on childhood fever after completing the baseline MCQ test (focused on fever definition, fever measurement, adverse effects management and situations requiring a physician)  Tests done by research group | Multiple choice based of previous studies (Casey, Robinson, Schmitt)  Test were all scenarios with MCQ responses and scored by the same person  MCQ was given at 3 times, baseline, 2 and 4 months after intervention. | Knowledge  Health Service utilization | - Test scores prior to intervention showed no statistical difference (p = 0.35), however subsequence test scores did reveal significant improvement of the intervention group over the control group at both time points (p = 0.006 at 2 months and p = 0.002 at 4 months) - No statistical difference between clinic and emergency room visits for appropriate and inappropriate fever management across the 2 group (p > 0.99) - Showed that parents with one child compared to other groups had a significantly higher number of inappropriate visits to the ER (p = 0.04) - No significant differences found between utilization patterns and status of the study member’s active duty. | Huge attrition bias only 31 finishing the study (17 interventions and 14 control)  Unbalance control to intervention group  Not random allocation  No blinding of assessors or personnel specified  **MOD RISK** |
| Wasunna B, et.al  (2010) (S37)  **Kenya** | Cross sectional study (pre-post) | Aimed to improve how artemisinin-based combination therapy (ACT) is administered to febrile children presenting to government clinic in a highly malaria endemic area of Kenya  Improving the performance of health workers at facilities, focusing on reinforcement of AL treatment for febrile children and adherence to recommended dispensing and counselling tasks in accordance with national guidelines | health working stations | Training healthcare workers in Kenya | **33 government health facilities took part**  **48 health care workers and 386 febrile children in baseline**  **36 health care workers and 390 febrile children for the intervention** | **Group training sessions:**  Done by nurses, lab technicians, public health officers, clinical officers and doctors.  Training took place over 3 days in the form of workshops. It consisted of many training units (specifically on malaria treatment, prevention, management and AL stocking. It also included practical sessions on diagnostic procedures, as well as a day devoted to managing uncomplicated malaria)  Each training unit was also covered in a manual which included objectives. Revised national guidelines, the stated training manuals, booklets and wall charts/posters were also given on the topic.  This was split into lectures, group discussions, role-plays, case scenario and questions and answers quizzes. | Surveys including an audit of government health facilities took place pre-and post-intervention (this focused on assessing drug stock, availability of equipment and training)  Health worker structured interview to obtain information on demographics and exposure to training and guidelines  Exit interviews of caretakers of sick children under 5 years old presenting to the outpatient department on the survey day.  Health care workers took a pilot of the training and gave feedback (changing the duration of the intervention from2 to 3 days) | Health service utilization | - Of 36 interviewed health workers who performed outpatient consultations for febrile children on survey days, 67% had received the intervention training and 61% had access to either a personal or facility copy of the national malaria case management guidelines. - At the 33 facilities, the most commonly observed job-aid was a wall chart with an algorithm for assessing and treating children below five years (76%), followed by the poster recommending presumptive treatment of childhood fevers with AL (58%) and AL dispensing wall chart (52%). - None of the health workers had received all components of the intervention and only 50% (18/36) were exposed to the minimum intervention package as defined above. - The proportion of febrile children having AL prescribed was relatively high during the pre-intervention survey, 76.9% (95% CI: 69.4-83.1), yet it increased to 87.6% (95% CI: 82.5-91.5) during the post-intervention survey. - There was an 18% increase in the numbers of children leaving the health facility with recommended AL during the post-intervention survey and none of the children who did not have AL prescribed left facility with AL dispensed. - During both surveys, nearly all (98.5% and 99.1% respectively) caretakers of children for whom AL was dispensed, reported that the health workers provided advice on completion of the AL treatment regimen. - During both surveys, few children (1.5% before and 5.0% after the intervention) received all four AL dispensing and counselling tasks while the performance of at least three out of four tasks increased from 20.1% (95% CI: 10.2-35.9) to 34.8% (95% CI: 21.0-52.1) although the difference was not statistically significant. - Despite minor improvements in areas, health care workers with enhanced in-service training, guidelines and job aids showed no statistically significant differences in any indicator compared to pre-intervention | Eligibility criteria not specified  No measurements for confounders  Pre-and post-intervention questionnaires were slightly different  Possible selective reporting  **MOD-HIGH RISK** |
