# Supplementary material for: Educational interventions on fever management in children: A scoping review
Source: Nurs Open. 2019 May 1;6(3):713–21. doi: 10.1002/nop2.294 (PMC6650695; doi:10.1002/nop2.294)
Supplement: Supplementary file 3 [file NOP2-6-713-s003.docx]

**List of Supplementary References**

1. Abbey M, Bartholomew LK, Pappoe M, Van den Borne B. Treating fever in children under 5 years of age: Caregiver perceptions of community health worker services in Dangme West district Ghana. *International Health.* 2015;7(6):455-63. doi:10.1093/inthealth/ihv027
2. Baker MD, Monroe KW, King WD, Sorrentino A, Glaeser PW. Effectiveness of fever education in a pediatric emergency department. *Pediatric Emergency Care.* 2009;25(9):565-8. doi:10.1097/PEC.0b013e3181b4f64e
3. Bloch SA, Bloch AJ. Using Video Discharge Instruction as an Adjunct to Standard Written Instructions Improved Caregivers’ Understanding of Their Child’s Emergency Department Visit, Plan, and Follow-up. *Pediatric Emergency Care.* 2013;29(6):699-704. doi:10.1097/PEC.0b013e3182955480
4. Broome ME, Dokken DL, Broome CD, Woodring B, Stegelman MF. A study of parent/grandparent education for managing a febrile illness using the CALM approach. *Journal of Pediatric Health Care.* 2003;17(4):176-83
5. Casey R, McMahon F, McCormick MC, Pasquariello PS Jr, Zavod W, King FH Jr. Fever Therapy: an educational intervention for parents. *Journal of Pediatrics.* 1984;73(5):600-5
6. Chang LC, Lee PI, Guo NW, Huang MC. Effectiveness of Simulation-Based Education on Childhood Fever Management by Taiwanese Parents. *Pediatrics and Neonatology.* 2016;57(6):467-473
7. Chibwana AI, Gomersall JS. Management of Febrile illness in children less than 5 years of age at Limbe Health Center, Blantyre District in Malawi: a best practice implementation project. *JBI Database of Systematic Reviews and Implementation Reports.* 2013;11(12):256-272
8. Chirdan OO, Zoakah AL, Ejembi CL. Impact of Health Education on Home Treatment and Prevention of Malaria in Jengre, North Central Nigeria. *Annals of African Medicine.* 2008;7(3):112-119
9. Considine J, Brennan D. Effect of an evidence-based education programme on ED discharge advice for febrile children. *Journal of Clinical Nursing.* 2007;16(9):1687-1694
10. Considine J, Brennan D. Effect of an evidence-based paediatric fever education program on emergency nurses’ knowledge. *Accidental Emergency Nursing.* 2007;15(1):10-19
11. Considine J, Brennan D. Emergency nurses’ opinion regarding paediatric fever: The effect of an evidence-based education program. *Australasian Emergency Nursing Journal.* 2006;9(3):101-111
12. Cropley L. The effect of health education interventions on child malaria treatment-seeking practices among mothers in rural refugee villages in Belize, Central America. *Health Promotion International.* 2004;19(4):445-452
13. Cunningham A, Edwards A, Jones KV, Bourdeaux K, Willock J, Barnes R. Evaluation of a service development to increase detection of urinary tract infections in children. *Journal of Evaluation in Clinical Practice.* 2004;11(1):73-76
14. De Vos Kerkhof E, Nijman R, Vergouwe Y, Polinder S, Steyerberg E, Van der Lei J, Moll HA, Oostenbrink R. Impact of a Clinical Decision Model for Febrile Children at Risk for a serious Bacterial Infection at the Emergency Department: A Randomized Controlled Trail. *PLoS One.* 2015;10(5)
15. Edwards H, Walsh A, Courtney M, Monagham S, Wilson J, Young J. Promoting evidence based childhood fever management through a peer education programme based on the theory of planned behaviour. *Journal of Clinical Nursing.* 2007;16(10):1966-79.
16. Edwards H, Walsh A, Courtney M, Monagham S, Wilson J, Young J. Improving paediatric nurses’ knowledge and attitudes in childhood fever management. *Journal of Advanced Nursing.* 2007;57(3):257-269
17. Eriksen J, Mujinja P, Warsame M, Msimba S, Kouyalé B, Gustafsson LL, Jahn A, Muller O, Sauerborn R, Tomson G. Effectiveness of a community intervention on malaria in rural Tanzania – a randomised control trial. *African health Sciences.* 2010;10(4):332-340
18. Fieldston E, Nadel F, Alpern E, Fiks A, Shea J, Alessandrini E. Effects of an education and training intervention on caregiver knowledge of nonurgent pediatric complaints and on child health services utilization. *Pediatric Emergency Care.* 2013;29(3):331-336
19. Hart L, Nedadur R, Reardon J, Sirizzotti N, et.al. Web-based tools of educating Caregivers and Childhood fever: A randomised Control Trial. *Pediatric Emergency Care.* 2016. Advance Online Publication. doi:10.1097/PEC.0000000000000936
20. Hu F, Zhang J, Shi S, Zhou Z. Fever Management in the emergency department of the Children’s hospital of Fudan University: A best practice implementation project. *JBI Database of systematic reviews and implementation reports.* 2016;14(9):358-366
21. Huang M-C, Liu C-C, Huang C-C. Effects of an educational program on parents with febrile convulsive children. *Pediatric Neurology.* 1998;18:150-155
22. Huang M-C, Liu C-C, Huang C-C, Chi Y-C, Thomas K. Effects of educational intervention on changing Parental Practices for recurrent febrile convulsion in Taiwan. *Epilepsia.* 2002;43(1):81-86
23. Ismail S, McIntosh M, Kalynych C, Joesph M, Wylie T, Butterfield R, Smotherman C, Kraemer DF, Osian SR. Impact of video discharge instruction for pediatric fever and closed head injury from the emergency department. *Journal of Emergency Medicine.* 2016;50(3):e177-e183
24. Jeong YS, Kim JS. Childhood fever management program for Korean pediatric nurses: A comparison between blended and face-to-face learning method. *Contemporary Nurse.* 2014;49:35-46
25. Kelly L, Morin K, Young D. Improving Caretakers’ knowledge of fever management in preschool children: Is it Possible? *Journal of Pediatric Health Care.* 1996;10(4):167-173
26. Light P, Hupcey J, Clark M. Nursing Triage and its influence on parents’ choice of care for febrile children. *Journal of pediatric Nursing.* 2005;20(6):424-429
27. Marsh VM, Mutemi WM, Muturi J, Haaland A, Watkins WM, Otieno G, Marsh K. Changing home treatment of childhood fevers by training shop keepers in rural Kenya. *Tropical Medicine and International Health.* 1999;4(5):383-389
28. O’Neill-Murphy K, Liebman M, Barnsteiner JH. Fever education: Does it reduce parent fever anxiety? *Pediatric Emergency Care.* 2001;17(1):47-51
29. Pusic MV, MacDonald WA, Eisman HO, Black JB. Reinforcing outpatient medical student learning using brief computer tutorials: The Patient-Teacher-Tutorial sequence. *BMC Medical Education.* 2012;12:70
30. Robinson J, Schwartz M-L, Magwene K, Krengel S, Tamburello D. The impact of fever Health Education on clinic utilization. *American Journal of Disease of Children.* 1989;143(6):698-704
31. Ruvinsky S, Mónaco A, Pérez G, Taicz M, et.al. Effectiveness of a program to improve antibiotic use in children hospitalized in a children’s tertiary care facility in Argentina. *Arch Argent Pediatr.* 2014;112(2):124-131
32. Sanghavi DM. Taking Well-child care into the 21^st^ Century: A novel, effective method for improving parent knowledge using computerized tutorials. *Arch Pediatr Adolesc Med.* 2005;159(5):482-485
33. Sarrell M, Kahan E. Impact of a single-session education program on parental knowledge of and approach to childhood fever. *Patient Education and Counselling.* 2003;51(1):59-63
34. Schriger D, Baraff L, Buller K, Shendrikar MA, et.al. Implementation of clinical guidelines via a computer charting system: Effect on the care of febrile children less than three years of age. *J Am Med Inform Assoc.* 2000;7(2):186-95
35. Statile A, Unaka N, Thomson J, Sucharew H, Gonzalez del Ray J, White C. Implementation of an innovative pediatric hospital Medicine Education Series. *Hospital Pediatrics.* 2016;6(3):151-156
36. Steelman J, Kotchmar G, Brehm W, Greenwall K. Childhood fever education in a military population: is education enough? *J Miss State Med Assoc.* 1999;40(12):407-9
37. Wasunna B, Zurovac D, Bruce J, Jones C, Webster J, Snow R. Health worker performance in the management of paediatric fevers following in-service training and exposure to job aids in Kenya. *Malaria Journal.* 2010;9:261
